# Outcomes following a behaviour change intervention within hospitals to improve birth registrations and hospital utilisation for Aboriginal and/or Torres Strait Islander infants: a quasi-experimental and cohort study

**DOI:** 10.1136/bmjopen-2024-089098

**Published:** 2025-10-20

**Authors:** K McAuley, Natalie Ann Strobel, Daniel Christensen, Karen Margaret Edmond, Peter Jacoby, Daniel McAullay

**Affiliations:** 1 Te Whatu Ora Health New Zealand Southern, Dunedin, Otago, New Zealand; 2 Kurongkurl Katitjin, Edith Cowan University, Perth, Western Australia, Australia; 3 King’s College London, London, England, UK; 4 Telethon Kids Institute, Perth, Western Australia, Australia

**Keywords:** Hospitalization, EPIDEMIOLOGY, Health Services, Community child health

## Abstract

**Objectives:**

The primary objective was to determine whether a behaviour change intervention delivered to hospital staff would (1) improve the proportion of Aboriginal and/or Torres Strait Islander (Aboriginal) babies being registered and (2) reduce hospital admissions and emergency presentations for babies <6 months old. The secondary objective was an observational analysis to determine factors that might influence the proportion of registered Aboriginal births in Western Australia (WA).

**Design:**

Quasi-experimental design and cohort study.

**Setting:**

Five tertiary birthing hospitals in WA.

**Participants:**

The intervention was delivered to health service providers who were in the five tertiary birthing hospitals. Outcome data were collected on Aboriginal babies born between 1 January 2016 and 30 June 2018 who were delivered within these hospitals. Babies in the control group (n=226) were born 6 months before the intervention and intervention babies (n=232) were born 6 months following the intervention. For the secondary objective, there were 4573 babies included in the analysis.

**Interventions:**

A behaviour change intervention delivered to hospital staff in five hospitals.

**Primary and secondary outcome measures:**

The primary outcomes were the proportion of babies who were registered and whether a baby had been admitted to hospital or an emergency department by 3 and 6 months old. The secondary outcome was to determine factors that might influence the proportion of registered Aboriginal births in WA (cohort study).

**Results:**

There was evidence of a 38% reduction in emergency presentations within 6 months for babies born to hospitals 6 months following the staff training (OR 0.62, 95% CI 0.42 to 0.91), and little evidence of improvements in birth registrations, hospital admissions within 3 or 6 months of birth or emergency department presentations within 3 months of birth. Of the 4573 babies included in the cohort study, 3769 (82.4%) babies had their births registered and 804 (17.6%) babies did not. Factors that were associated with not having a birth registered included low birth weight babies with a 34% decrease in odds of having a registered birth compared with those with a normal birth weight (adjusted OR (aOR) 0.66, 95% CI 0.51 to 0.86). Timing of first antenatal visit was associated with reduced odds of having a birth registered if this occurred in the second (aOR 0.77, 95% CI 0.64 to 0.93) or third trimester (aOR 0.59, 95% CI 0.45 to 0.77) compared with the first trimester.

**Conclusions:**

Our study identifies the complexities surrounding birth registrations and improved hospital utilisation for Aboriginal babies, the importance of targeted interventions and ongoing efforts needed to address this issue comprehensively.

**Trial registration number:**

ACTRN12615000976583.

**STRENGTHS AND LIMITATIONS OF THIS STUDY**

- This study highlighted the difficulties in delivering large-scale stepped-wedge cluster-randomised controlled trials (RCTs) within Western Australia due to the removal of babies by child protection from mothers during their time in hospital post partum and significant barriers to implementing multisite hospital research due to extensive governance approvals.
- Although we were able to complete a quasi-experimental design in lieu of the stepped-wedge cluster-RCT, there are a number of limitations to this study design including lack of randomisation and small sample size.
- Although the intervention supported behaviour change in health service providers, we did not directly measure their behaviour change.

## Introduction

 The health of Australian Aboriginal and Torres Strait Islander (hereafter referred to as Aboriginal) babies continues to be of concern despite substantial investments from federal and state governments.^1^,^3^ Compared with non-Aboriginal children, Aboriginal babies have the highest hospitalisation rates and are most in need of primary care health services during the first 6 months of life.^4^,^7^ Previous research has shown that up to 70% of Aboriginal children present to the emergency department and 40% are hospitalised, at least once, within their first year of life, with an increased risk within the neonatal (0 to <1 month) period and for preterm babies.^4 5^ Culturally safe maternity services with continuity of care into the postpartum period have the potential to improve the health of Aboriginal babies and into childhood.^8^,^11^

Concerns have also been expressed over delays in registering births or the lack of registration for Aboriginal babies, and the impact this can have on families in receiving appropriate services. In Western Australia (WA), a study of Aboriginal births between 1980 and 2010 revealed that 11% of births were unregistered,^12^ with a variation in the proportion unregistered by year. Factors found to have a strong association with not registering a birth included young maternal age at first birth, remoteness, a mother herself being unregistered and no private health insurance.^12^ Lack of birth registration is not unique to WA, with other Australian jurisdictions also seeing a delay or lack of birth registration for Aboriginal babies.^13 14^

Targeted models of early infant primary care (including home visiting, family-centred primary healthcare and educational interventions) have been developed and evaluated within Australia, and internationally, with positive outcomes for improving child health.^15^,^21^ Limitations of these models include restricted capacity to up-scale, issues with cost-effectiveness and sustainability, and lack of testing within complex multiorganisation environments.^15^,^21^ Difficulties also remain with contacting those most disadvantaged and families who move between jurisdictions. Models of care have also struggled to overcome communication issues between primary care providers, especially when families are using multiple services. The use of electronic health records can assist with some of these issues; however, as well as benefits for the most marginalised and disadvantaged, the use of such systems does not come without concerns around privacy and confidentiality.^22 23^

Enhancing care coordination through maternity hospitals has the potential to reduce barriers and improve the provision of early infant primary care.^24^,^27^ Existing models currently struggle to provide care for the most mobile families and there is a need to improve choice in primary care providers for families.^28 29^

In 2015, we registered a cluster-randomised controlled trial (RCT) to improve access to primary care and the health of Aboriginal babies under 3 months of age in WA.^30^ We developed an enhanced model of targeted support and early infant primary care coordination from hospital into primary healthcare for Aboriginal babies. Our original model of care was to recruit mothers/families within the birth hospital; however, there were complications in recruiting mothers immediately post partum, and active recruitment of mothers was ceased. As a result, we embarked on an interview process with health service providers, with feedback from research staff, to enable a behaviour change intervention that focused on discharge planning, providing information to mothers and improving standard practice by health service providers. As a result, all mothers who delivered a baby in an intervention hospital following the interview and feedback process were anticipated to benefit from the intervention.

The primary objectives of this study were to determine whether a behaviour change intervention delivered to hospital staff would (1) improve the proportion of Aboriginal babies being registered and (2) reduce hospital admissions and emergency presentations for babies <3 months and <6 months old. The secondary objective was an observational analysis to determine the factors that might influence the proportion of registered Aboriginal births in WA for babies born between 1 January 2016 and 30 June 2018.

## Methods

### Original study

The original study to determine the effectiveness of a targeted support and care coordination intervention delivered to Aboriginal mothers has been previously described.^30^ The study was initially designed to be a stepped-wedge, cluster-RCT. 25 hospitals were placed into 22 geographical locations. These 22 locations were randomised to determine which hospitals were included in the five clusters. Each step was a 6-month time period, with the first 6 months a baseline period. All government public birthing hospitals in WA with five or more Aboriginal births per year were to receive the intervention in a stepwise (staggered) roll-out over a 30-month period. Based on our protocol, we had anticipated that approximately 1900 Aboriginal infants and their mothers born over a 30-month period would be involved in the intervention. In total, approximately 4300 Aboriginal infants would be followed up from birthing hospitals.

During the pilot study in September 2016, we faced significant issues in implementing the intervention as described in our published protocol.^30^ These included recruiting mothers during their hospital stay, including babies being removed from their mothers after their birth by child protection and mothers leaving hospital 6 hours after birth. In addition, it became clear it was impossible to implement multiple hospital research in WA due to governance approvals for each hospital to participate. Due to the difficulties in receiving governance approval within WA hospitals and issues in recruiting mothers directly post partum, delivering the intervention to all hospitals within the initial 24-month period was found to be not feasible. As a result, we made the following changes to the original protocol. The study design changed from a stepped-wedge cluster-RCT to a quasi-experimental design. Details of this new study design are described under the section ‘Study design’. Rather than mothers being recruited to the intervention, health service providers were instead recruited who then delivered the intervention. This also changed the type of intervention that we delivered; however, the aim of the intervention remained the same (details provided under ‘Intervention and control groups’).

### Study design

We used a quasi-experimental design to determine the effectiveness of the intervention. We have used the quasi-experimental checklist to help define the features of the study (online supplemental appendix A).^31^ The study was implemented from 1 January 2016 to 30 June 2018.

To determine the proportion of registered Aboriginal births and factors that influenced registration across WA, a birth cohort for babies born between 1 January 2016 and 30 June 2018, excluding those babies that received the intervention, was used.

### Study setting

This study was conducted across WA, which has a population of 2.8 million people and covers a geographical area of 2.5 million km^2^.^32^ It has the most remote regions in Australia, with 95% classified as Australian Standard Geographical Classification Remoteness Structure level 4 (remote) and 5 (very remote). Approximately 40% of the Aboriginal population in WA resides within these remote and very remote regions.^33^ In 2015, there were 34 757 live births, and of these, 1731 births were Aboriginal babies.^34^ 37 birthing hospitals are located in WA: 28 public (1 tertiary maternity hospital and 27 secondary hospitals) and 9 private birthing hospitals. We excluded hospitals that had <5 births/year; therefore, 25 hospitals were included in this study.

### Hospital selection

**Figure 1:**
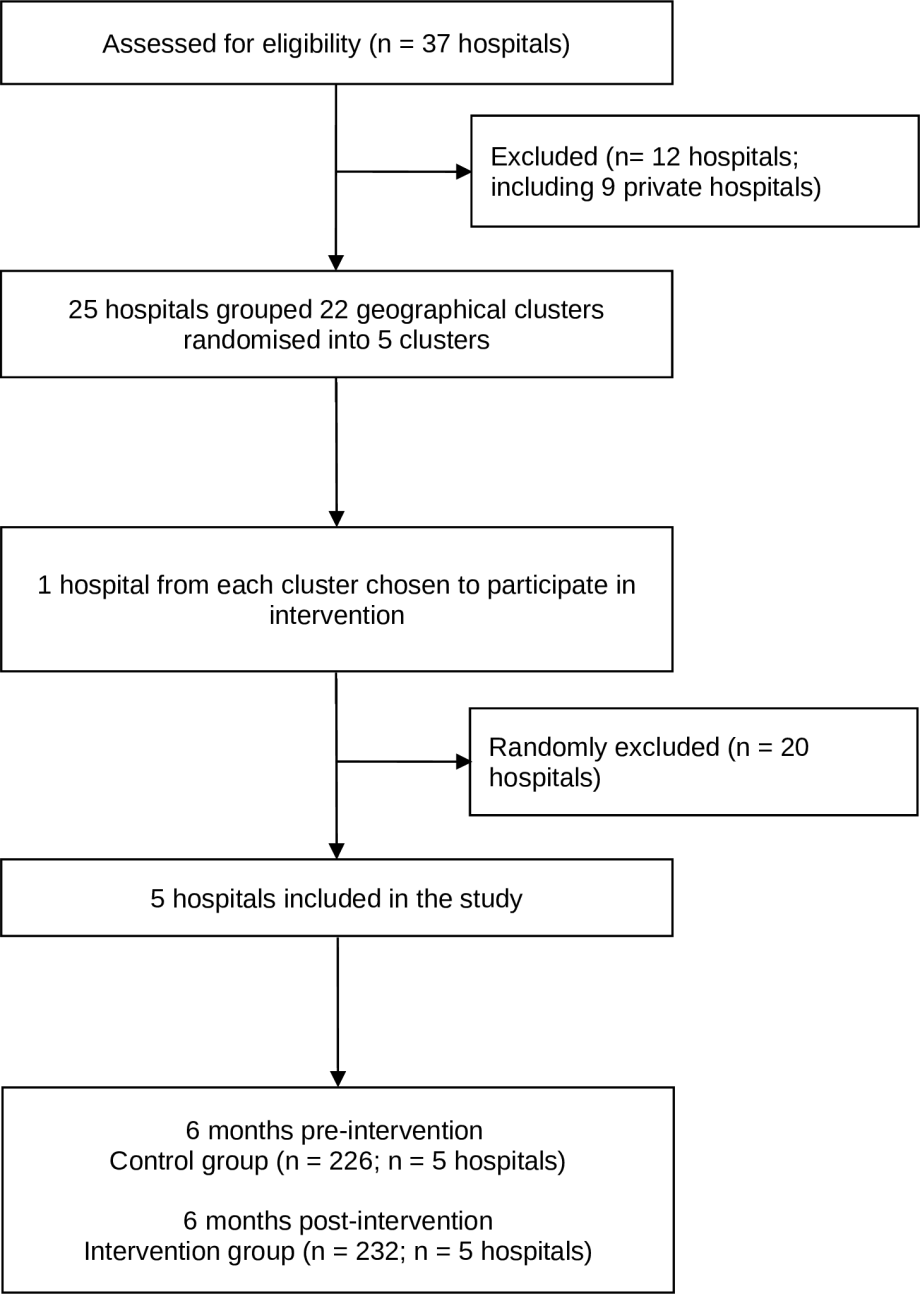
Study flow diagram.

2 hospitals were allocated to 22 distinct geographical areas of birthing hospitals and were the unit of randomisation. As per the original protocol, the 22 clusters were labelled between 1 and 22. A separate Excel spreadsheet was created with a column containing the numbers 1 to 22, and using a random number generator, a number was generated from 0 to 1 next to each number in the column containing the de-identified cluster data (numbers 1 to 22). The two columns were then sorted by the column containing the random number, from the smallest to the largest. Clusters were allocated as per the original protocol. Hospitals were unaware of their allocation to each cluster. As part of the new study design, a research team member approached the first hospital listed per cluster to be involved in the intervention. If a hospital declined or there was no response to our request, we then approached the next hospital in line in that cluster. Directors and managerial staff from the intervention hospitals were approached individually with information about the study to gain their support and secure participation. Final approval was granted by hospital directors for participation in the study and individual consent from health service providers was sought. As a result, a total of five hospitals, one from each cluster, participated in the intervention (figure 1; online supplemental appendix B).

### Intervention and control groups

**Figure 2:**
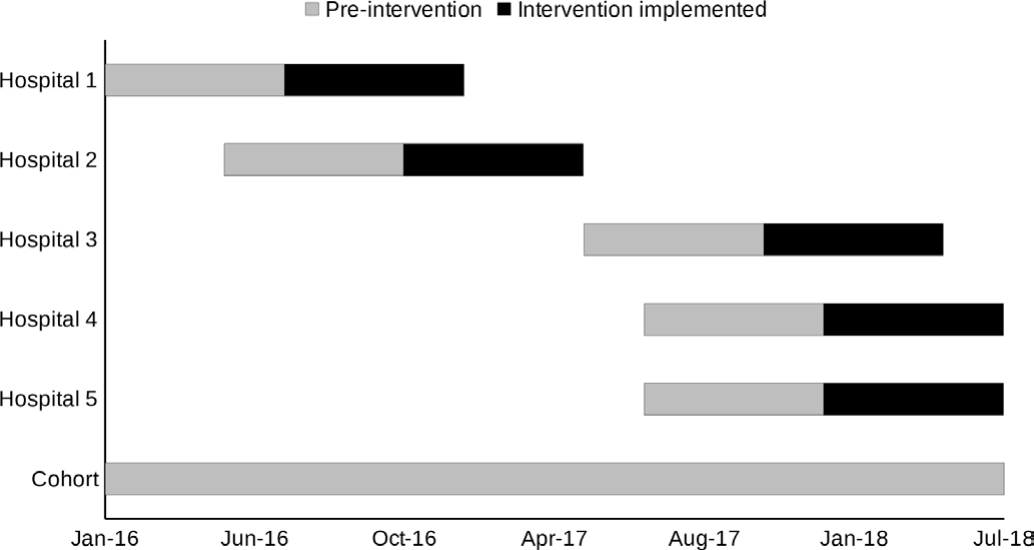
Timing of the study across control and intervention sites.

The intervention was delivered between June 2016 and July 2018. The intervention was defined as commencing the first day of the month that interviews were completed and the following six full calendar months (figure 2). Babies born 1 day before the intervention and the preceding six calendar months in each intervention hospital were part of the control group.

Based on feedback from hospital staff who participated in the pilot study, it was determined that an interview process with health service providers, and feedback from research staff, enabled behavioural change in discharge planning, information provided to mothers and standard practice among the health service providers. As a result, we revised our intervention to be a capacity-building training programme for hospital midwives, nurses and maternity hospital staff, designed to improve their skills and confidence in providing discharge planning support for new mothers. The expected mechanism of change was that better-trained nurses will deliver higher quality, more consistent discharge education and planning, which will in turn influence mothers’ health knowledge, self-efficacy and post-discharge care behaviours. The population within the intervention was defined as:

- Intervention recipients: midwives, nurses and maternity hospital staff whose knowledge, attitudes or practices that we were directly trying to change.
- Intervention beneficiaries: new mothers and their Aboriginal babies who should receive improved discharge planning and follow-up care as a result of the staff’s new skills.

To determine whether there was evidence of behaviour change in the beneficiaries (ie, mother-baby dyads) of the intervention and possibly better follow-up care as a result of intervention recipients’ new skills, we assessed the impact of the intervention on the intervention beneficiaries (see Outcomes).

To complete this process, we interviewed health service staff within the five hospitals, and if there were local Aboriginal Community Controlled Health Organisations (ACCHOs) (Indigenous governed primary healthcare services) that supported these hospitals, then these staff were also interviewed. Semistructured interview questions included:

1. What do you consider is best practice in discharge planning, post-natal care and health education?
2. What is your current practice in discharge planning, post-natal care and health education?
  - What information do you give to families after birth?
  - Is there anything that is missing in this information?
3. Is there anything that is preventing you from achieving best practice in discharge planning, post-natal care and health education?
4. Is there anything that will assist you to achieve best practice in discharge planning, post-natal care and health education?

Using NVivo, data from these interviews were analysed into four themes: current practice, barriers, enablers and recommendations. Staff were provided with a report that covered these four themes with an example of a report provided in online supplemental appendix C. Researchers provided hospital staff with a 30 min online presentation of the results and the written report. Staff then undertook continuous quality training (CQI) with a trained facilitator to improve the delivery of care to mothers through plan-do-study-act (PDSA) cycles. The CQI training included a 1-hour webinar which was an overview and introduction to CQI. This was followed by 2×1.5 hours face-to-face practical workshops that involved working through a PDSA cycle, using an issue or problem relevant to their organisation. The workshop used data examples presented from the report to help set up a PDSA cycle. Overall, participants were taken through the following subject matter throughout the CQI training: organisational culture for CQI, leadership, systems thinking, population health approach, client-centred care, workforce, resources, systematic use of data, tools (PDSA), teamwork, external partnerships and relationships and community engagement. This process was undertaken with all intervention hospitals with the aim of facilitating behaviour change in healthcare workers who had contact with mothers during and soon after birth. Depending on the reports provided to hospitals, change may have occurred through mother knowledge (eg, breastfeeding, importance of immunisations, importance of well-child checks, importance of hygiene), facilitation of engagement with primary care (eg, asking mother who she might like to be seen by) and informing the primary care practice of choice allowing for primary healthcare providers to follow up if they did not hear from the mother. We have developed an indicative programme logic to illustrate one example of the types of change the intervention sought to achieve (online supplemental appendix D). This example is intended to demonstrate the underlying theory of change, acknowledging that specific activities and intended outcomes vary across different implementation settings. The intervention is designed to be flexible so that activities can address context-specific service gaps and local needs while remaining aligned with the overall research programme goals. However, as part of the intervention, we did not collect data on what activities the health service providers undertook and the time they spent with mothers. All mothers who delivered in the intervention hospital following the interview and feedback process were anticipated to benefit from the intervention. We assumed that changes within the hospital process would occur over 6 months and as such considered changes to the intervention during this period (figure 2).

### Participants

Participants were babies born between 1 January 2016 and 30 June 2018 who were classified as Indigenous using the Department of Health’s derived Aboriginal and Torres Strait Islander Status Flag, which combines several sources of data to come up with an overall derived Aboriginal and Torres Strait Islander status of ‘Yes’, ‘No’ or ‘Missing’, for that individual.^35^ The present study was composed of babies with status of ‘yes’, excluding stillbirths.

### Outcomes

The outcomes were binary (yes/no) measures for birth registrations, hospital admissions and emergency department presentations. Birth registration records only include those babies where the parent/s have lodged an application to register the birth.^12^ There was no time limit for when a birth was registered for babies to be included as a registered birth and only the year of registration is provided. Birth registrations of babies were provided up to 31 December 2019, 18 months after the end of the study. Babies who died within the 6 month pre-intervention or post-intervention were excluded from the analyses (n=4). In Australia, it is a requirement within each state that a birth is registered within 60 days of delivery, though a registration can be completed beyond this time period.^36 37^

For this study, a hospital admission was defined as an all-cause admission to any Western Australian hospital ward. This excluded the normal hospital stay after birth for well babies. An emergency department presentation was defined as any presentation to an emergency department regardless of whether the child was admitted to hospital. Both hospitalisation and emergency presentations were measured at <3 months and <6 months old.

### Covariates

There were 11 covariates identified, which were used to determine their association with birth registration. These included date of birth, infant’s recorded sex, gestational age, birth weight, plurality, parity, mother’s age at birth of child, any complications of pregnancy, the estimated gestational age of the child in weeks at the first antenatal visit, the area level Index of Relative Socio-Economic Disadvantage and the remoteness index.

All covariates used were derived from the Midwives’ Notification System. The infant’s sex is recorded by the midwife. Gestational age at birth was categorised as <37 or ≥37 weeks. Birth weight was categorised as either <2500 g or ≥2500 g. Plurality refers to multiple births during a single pregnancy. Parity defines the total number of previous pregnancies resulting in a birth of one or more babies where the pregnancy was at least 20 weeks’ gestation. For the purpose of this publication, we divided parity into <3 previous births or ≥3 previous births. Maternal age at birth was categorised as <19 years or ≥20 years. Complications of pregnancy included conditions such as threatened miscarriage, threatened pre-term labour, pre-eclampsia, antepartum haemorrhage, pre-labour rupture of membranes, gestational diabetes, gestational hypertension or other International Statistical Classification of Diseases and Related Health Problems 10th Revision (ICD10-AM) codes. A mother was considered to have a complication of pregnancy if any of the complications were listed. The first antenatal visit was categorised into trimesters using estimated gestational age (1–13 weeks, 14–27 weeks, ≥27 weeks). Area level Index of Relative Socio-Economic Disadvantage and the remoteness index, both from the 2016 Australian Bureau of Statistics’ Census of Population and Housing, were based on the mother’s address at the birth of the child. Area level disadvantage was categorised as state quintiles from most disadvantaged (1) to least disadvantaged (5). Remoteness area classifies geographic location on the basis of isolation and distance from service centres and healthcare facilities. These data were split into four categories from least remote (major cities) to most remote (very remote areas).

### Data collection

This study used linked data from the Midwives’ Notification System, Death Registrations and Birth Registrations. The databases were systematically linked by the Data Linkage Branch from the WA Department of Health using probabilistic matching and de-identified records.^38^

The Midwives’ Notification System includes clinical data (infant weight, gestational age, parity, complications of pregnancy) and sociodemographic data (mother’s age, Indigenous status, socioeconomic status, remoteness index) for all Western Australian live births and stillbirths of more than 20 weeks’ gestation or birth weight >400 g. Births are reported by trained midwives within 48 hours of delivery. Birth and death registrations are supplied by the Registry of Births, Deaths and Marriages. Only the year of birth registration was provided for this study.

### Data analysis

Analyses were conducted to (1) estimate the evidence of an effect of the intervention on birth registrations, hospital admissions and emergency presentations and (2) determine the proportion of births registered in a larger cohort and factors associated with an infant’s registration. A different analytic sample was used for each analysis.

To estimate evidence of an intervention effect, conditional logistic regression was used to remove any time-invariant (fixed) measured or unmeasured effects at the hospital level, using the *clogit* procedure in Stata. As data were aggregated for all hospitals other than the intervention sites, intervention analyses were restricted to the five intervention sites. Given no substantial differences at baseline between the intervention and control groups, we did not adjust the analyses. To investigate whether the intervention was affected by outside policy, we completed a sensitivity analysis through an investigation of the secular trend of hospital admissions and emergency presentations in a cohort of babies born between 1 January 2016 and 30 June 2018 (n=4347) in hospitals within WA that were not included in the intervention. We did not complete this analysis for birth registrations as we were only provided with year of registration and not exact dates. We completed an additional sensitivity analysis for all outcomes through an investigation of a 1-month washout period of the intervention to determine whether there was any cross-contamination during the transition between the control and the intervention time periods. We also investigated whether there were seasonal changes that may influence hospital admissions and emergency presentations. In particular, we investigated whether winter may have influenced hospital utilisations as many infants experience respiratory infections (eg, Respiratory syncytial virus, influence) during this time. We created a proxy binary variable that was defined as babies who had the opportunity to present to emergency during winter if winter was within 0–92 days of their date of birth (3 months) or within 0–184 days of their date of birth (6 months). Due to sample size, we were only able to create a binary variable. This approach also controlled for any imbalances between the intervention and control groups in their exposure to hospital and emergency presentation risks due to winter.

Logistic regression was used to estimate the unadjusted and adjusted associations between the covariates and birth registration prior to 1 January 2020. A directed acyclic graph (DAG) was used to assess potential confounding relationships on birth registration (online supplemental appendix E). Based on the DAG, a minimal sufficient adjustment set to de-confound each exposure was identified in DAGitty V.3.0.^39^ We excluded year of birth from the analysis as we are unable to provide the period of time that all babies had the opportunity to be registered. To exclude the effect of the intervention, the analysis consisted of all babies born in the control arm plus those that were included in the sensitivity analysis outlined above (n=4573) in hospitals within WA that were not included in the intervention.

No imputation was undertaken and missing data were excluded from all analyses, as missing data were negligible (<6%), with missing cell counts specified within all the tables. Data were missing due to either the non-completion of administrative data or (in the case of area disadvantage or remoteness data) address data which could not be coded. Small cell counts (n≤5) have been suppressed for confidentiality.

### Patient and public involvement

The original intent was to involve mothers during the pilot phase of the project to inform the main study. However, due to issues that were experienced in the pilot phase, it was decided not to include mothers in the design and conduct of the study. Throughout the study, we did collaborate and were partners with ACCHOs who helped to inform the design, conduct and dissemination of the research. This included working with staff to design the new intervention and interviewing staff to help hospitals understand the barriers and facilitators that were experienced by families within organisations. Information from each intervention report was fed back to relevant ACCHOs.

## Results

### Effect of the intervention

There were 458 babies included in determining whether there was evidence of an effect of the intervention (table 1). Of these, 232 (50.7%) babies were born during the intervention and were included in the intervention group, and 226 (49.3%) babies in the control group (figure 1). The sample size for each intervention hospital by birth registrations, hospital admissions and emergency presentations is provided in online supplemental appendices B, F and G.

**Table 1:** Characteristics of births born before and after the intervention

Characteristics	Total number of infants n=458	Control n=226 (49.3%)	Intervention n=232 (50.7%)
*Infant*			
Sex			
Male	233 (50.9%)	115 (50.9%)	118 (50.9%)
Female	225 (49.1%)	111 (49.1%)	114 (49.1%)
Gestational age at birth			
<37 weeks old	50 (10.9%)	24 (10.6%)	26 (11.2%)
≧37 or more weeks	408 (89.1%)	202 (89.4%)	206 (88.8%)
Birth weight			
<2500 g	34 (7.4%)	18 (8%)	16 (6.9%)
≧2500 g	424 (92.6%)	208 (92%)	216 (93.1%)
Year of birth			
2016	217 (47.4%)	134 (59.3%)	83 (35.8%)
2017	152 (33.2%)	92 (40.7%)	60 (25.9%)
2018	89 (19.4%)	0 (0.0%)	89 (38.4%)
*Mother*			
Parity			
<3 previous births	113 (24.7%)	58 (25.7%)	55 (23.7%)
≧3 previous births	345 (75.3%)	168 (74.3%)	177 (76.3%)
Maternal age at birth			
<20 years old	67 (14.6%)	30 (13.3%)	37 (15.9%)
≧20 years old	391 (85.4%)	196 (86.7%)	195 (84.1%)
Any complications of pregnancy			
No	356 (77.7%)	178 (78.8%)	178 (76.7%)
Yes	102 (22.3%)	48 (21.2%)	54 (23.3%)
Estimated gestational age at first antenatal visit*			
First trimester	298 (65.1%)	148 (65.5%)	150 (64.7%)
Second trimester	114 (24.9%)	58 (25.7%)	56 (24.1%)
Third trimester	39 (8.5%)	18 (8%)	21 (9.1%)
*Area*			
Area socioeconomic index quintiles			
1 (most disadvantaged)	82 (17.9%)	41 (18.1%)	41 (17.7%)
2	62 (13.5%)	37 (16.4%)	25 (10.8%)
3	74 (16.2%)	36 (15.9%)	38 (16.4%)
4	128 (27.9%)	64 (28.3%)	64 (27.6%)
5 (least disadvantaged)	112 (24.5%)	48 (21.2%)	64 (27.6%)
Remoteness area			
Major cities	109 (23.8%)	44 (19.5%)	65 (28.0%)
Regional	121 (26.4%)	68 (30.1%)	53 (22.8%)
Remote	190 (41.5%)	93 (41.2%)	97 (41.8%)
Very remote	38 (8.3%)	21 (9.3%)	17 (7.3%)

* Missing data for seven children.

There were no notable differences in characteristics between the intervention and control babies (table 1). Overall, 11.2% (n=26) of babies in the intervention sites were preterm births compared with 10.6% (n=24) in the control group. 28% (n=65) of intervention babies were from major cities compared with 19.4% (n=44) of babies from the control group.

There was evidence of a 38% reduction in emergency presentation within 6 months of birth for babies who were in hospital during the intervention period (OR 0.62, 95% CI 0.42 to 0.91; table 2). There was little evidence of an effect of the intervention on the proportion of births registered (OR 0.89, 95% CI 0.54 to 1.46), hospital admission within 3 months (OR 0.74, 95% CI 0.46 to 1.20) and 6 months (OR 0.84, 95% CI 0.54 to 1.29) and emergency department presentations within 3 months of birth (OR 0.78, 95% CI 0.53 to 1.15).

**Table 2:** Conditional logistic regression on the effect of the intervention on birth registration, hospital admissions and emergency department presentations

	Total number of children	Number of children with an event (%)	Unadjusted OR (95% CI)	P value
*Birth registration*				
Control	226	191 (84.5%)	Ref.	
Intervention	232	193 (83.2%)	0.89 (0.54 to 1.46)	0.638
*Hospital admissions within 3 months of birth*				
Control	226	45 (19.9%)	Ref.	
Intervention	232	36 (15.5%)	0.74 (0.46 to 1.20)	0.227
*Hospital admissions within 6 months of birth*				
Control	226	58 (25.7%)	Ref.	
Intervention	232	52 (22.4%)	0.84 (0.54 to 1.29)	0.426
*Emergency department presentations within 3 months of birth*				
Control	226	95 (42%)	Ref.	
Intervention	232	84 (36.2%)	0.78 (0.53 to 1.15)	0.208
*Emergency department presentations within 6 months of birth*				
Control	226	141 (62.4%)	Ref.	
Intervention	232	117 (50.4%)	0.62 (0.42 to 0.91)	0.014

There is no evidence of any secular trends affecting hospitalisations or emergency department presentations within this sample (online supplemental appendix H). There is no evidence of an effect of the 1-month washout period of the intervention on hospitalisations, emergency department presentations or birth registrations (online supplemental appendix I). After adjusting for the seasonal variable, the evidence of an effect did not change for hospital admissions and emergency presentations (online supplemental appendix I).

### Observational analysis of factors influencing birth registration

There were 4573 babies included in determining what factors were associated with birth registration across WA between 1 January 2016 and 30 June 2018. Overall, there were 3769 (82.4%) of births registered and 804 (17.6%) of births that were not registered (table 3).

**Table 3:** Characteristics by birth registration

Characteristics	Total infants n=4573	Infants without registered births n=804 (17.6%)	Infants with registered births n=3769 (82.4%)
*Infant*			
Sex			
Male	2348 (51.3%)	405 (50.4%)	1943 (51.6%)
Female	2225 (48.7%)	399 (49.6%)	1826 (48.4%)
Plural birth			
No	4428 (96.8%)	778 (96.8%)	3650 (96.8%)
Yes	145 (3.2%)	26 (3.2%)	119 (3.2%)
Gestational age at birth			
<37 weeks old	695 (15.2%)	132 (16.4%)	563 (14.9%)
≧37 or more weeks	3878 (84.8%)	672 (83.6%)	3206 (85.1%)
Birth weight			
<2500 g	639 (14.0%)	142 (17.7%)	497 (13.2%)
≧2500 g	3934 (86.0%)	662 (82.3%)	3272 (86.8%)
Year of birth			
2016	1877 (41%)	294 (36.6%)	1583 (42%)
2017	1814 (39.7%)	325 (40.4%)	1489 (39.5%)
2018	882 (19.3%)	185 (23.0%)	697 (18.5%)
*Mother*			
Parity			
<3 previous births	3362 (73.5%)	500 (62.2%)	2862 (75.9%)
≧3 previous births	1211 (26.5%)	304 (37.8%)	907 (24.1%)
Maternal age at birth			
<20 years old	675 (14.8%)	102 (12.7%)	573 (15.2%)
≧20 years old	3898 (85.2%)	702 (87.3%)	3196 (84.8%)
Any complications of pregnancy			
No	3130 (68.4%)	566 (70.4%)	2564 (68.0%)
Yes	1443 (31.6%)	238 (29.6%)	1205 (32.0%)
Estimated gestational age at first antenatal visit*			
First trimester	2454 (53.7%)	393 (48.9%)	2061 (54.7%)
Second trimester	1415 (30.9%)	248 (30.8%)	1167 (31.0%)
Third trimester	444 (9.7%)	99 (12.3%)	345 (9.2%)
Area socioeconomic index quintiles^†^			
1 (most disadvantaged)	936 (20.5%)	252 (31.3%)	684 (18.1%)
2	972 (21.3%)	217 (27.0%)	755 (20.0%)
3	971 (21.2%)	150 (18.7%)	821 (21.8%)
4	825 (18.0%)	89 (11.1%)	736 (19.5%)
5 (least disadvantaged)	851 (18.6%)	94 (11.7%)	757 (20.1%)
Remoteness area^†^			
Major cities of Australia	1988 (43.5%)	243 (30.2%)	1745 (46.3%)
Regional Australia	996 (21.8%)	153 (19.0%)	843 (22.4%)
Remote Australia	548 (12.0%)	98 (12.2%)	450 (11.9%)
Very remote Australia	1023 (22.4%)	308 (38.3%)	715 (19.0%)

* Missing data for 260 children

† Missing data for 18 children

Babies with a low birth weight (497; 77.8%) had a 34% decrease in odds of having a registered birth compared with those with a normal birth weight (3272; 83.2%) (adjusted OR (aOR) 0.66, 95% CI 0.51 to 0.86; table 4). Having three or more previous births resulted in having a decreased odds in having a birth registered compared with having <3 previous births (aOR 0.52, 95% CI 0.44 to 0.61). Mothers aged <20 years old had 32% increased odds of registering their baby’s birth compared with mothers ≧20 years old (aOR 1.32, 95% CI 1.05 to 1.67). Timing of first antenatal visit was associated with reduced odds of having a birth registered if this occurred in the second (aOR 0.77, 95% CI 0.64 to 0.93) or third trimester (aOR 0.59, 95% CI 0.45 to 0.77) compared with the first trimester. Babies living in the least disadvantaged socioeconomic quintile had greater odds of having their birth registered compared with those living in the most disadvantaged quintile (aOR 2.03, 95% CI 1.54 to 2.69). Compared with living in the metropolitan area, living in regional (aOR 0.77, 95% CI 0.62 to 0.95), remote (aOR 0.64, 95% CI 0.49 to 0.83) or very remote (OR 0.32, 95% CI 0.27 to 0.39) WA was also associated with decreased odds of registering a birth.

**Table 4:** Differentiation between characteristics by birth registration

Characteristics	Total infants n=4573		Infants without registered births n=804 (17.6%)	Infants with registered births n=3769 (82.4%)	OR (95% CI)	P value	aOR (95% CI)	P value	Covariates (online supplemental appendix E)
*Infant*									
Sex									
Male	2348		405 (17.2%)	1943 (82.8%)	1.05 (0.90 to 1.22)	0.544	1.05 (0.90 to 1.22)	0.544	No adjustment
Female	2225		399 (17.9%)	1826 (82.1%)	1.00 (ref.)				
Plural birth									
No	4428		778 (17.6%)	3650 (82.4%)	1.00 (ref.)		1.00 (ref.)		Maternal age at birth
Yes	145		26 (17.9%)	119 (82.1%)	0.98 (0.63 to 1.50)	0.911	0.99 (0.64 to 1.52)	0.959	
Gestational age at birth									
<37 weeks old	695		132 (19.0%)	563 (81.0%)	0.89 (0.73 to 1.10)	0.289	0.87 (0.69 to 1.08)	0.209	Area socioeconomic index, maternal age at birth, plural birth, remoteness area, sex
≧37 or more weeks	3878		672 (17.3%)	3206 (82.7%)	1.00 (ref.)		1.00 (ref.)		
Birth weight									
<2500 g	639		142 (22.2%)	497 (77.8%)	0.71 (0.58 to 0.87)	0.001	0.66 (0.51 to 0.86)	0.002	Area socioeconomic index, gestational age at birth, maternal age at birth, parity, plural birth, remoteness area, sex
≧2500 g	3934		662 (16.8%)	3272 (83.2%)	1.00 (ref.)		1.00 (ref.)		
*Mother*									
Parity									
<3 previous births	3362		500 (14.9%)	2862 (85.1%)	1.00 (ref.)		1.00 (ref.)		Area socioeconomic index, maternal age at birth, remoteness area
≧3 previous births	1211		304 (25.1%)	907 (74.9%)	0.52 (0.44 to 0.61)	<0.001	0.52 (0.44 to 0.61)	<0.001	
Maternal age at birth									
<20 years old	675		102 (15.1%)	573 (84.9%)	1.23 (0.98 to 1.55)	0.068	1.32 (1.05 to 1.67)	0.017	Area socioeconomic index, remoteness area
≧20 years old	3898		702 (18.0%)	3196 (82.0%)	1.00 (ref.)		1.00 (ref.)		
Any complications of pregnancy									
No	3130		566 (18.1%)	2564 (81.9%)	1.00 (ref.)		1.00 (ref.)		
Yes	1443		238 (16.5%)	1205 (83.5%)	1.12 (0.95 to 1.32)	0.190	1.13 (0.67 to 1.34)	0.144	Birth weight, gestational age at birth, plural birth, sex
Estimated gestational age at first antenatal visit*									
First trimester	2454		393 (16%)	2061 (84%)	1.00 (ref.)		1.00 (ref.)		Area socioeconomic index, complication of pregnancy, maternal age at birth, parity, remoteness area
Second trimester	1415		248 (17.5%)	1167 (82.5%)	0.90 (0.75 to 1.07)	0.223	0.77 (0.64 to 0.93)	0.005	
Third trimester	444		99 (22.3%)	345 (77.7%)	0.66 (0.52 to 0.85)	0.001	0.59 (0.45 to 0.77)	<0.001	
Area socioeconomic index quintiles^†^									
1 (most disadvantaged)	936		252 (26.9%)	684 (73.1%)	1.00 (ref.)		1.00 (ref.)		Remoteness area
2	972		217 (22.3%)	755 (77.7%)	1.28 (1.04 to 1.58)	0.020	0.92 (0.74 to 1.15)	0.477	
3	971		150 (15.4%)	821 (84.6%)	2.02 (1.61 to 2.53)	<0.001	1.43 (1.13 to 1.82)	0.003	
4	825		89 (10.8%)	736 (89.2%)	3.05 (2.34 to 3.96)	<0.001	2.05 (1.55 to 2.72)	<0.001	
5 (least disadvantaged)	851		94 (11.0%)	757 (89.0%)	2.97 (2.29 to 3.84)	<0.001	2.03 (1.54 to 2.69)	<0.001	
Remoteness area^†^									
Major cities of Australia	1988		243 (12.2%)	1745 (87.8%)	1.00 (ref.)		1.00 (ref.)		No adjustment
Regional Australia	996		153 (15.4%)	843 (84.6%)	0.77 (0.62 to 0.95)	0.017	0.77 (0.62 to 0.95)	0.017	
Remote Australia	548		98 (17.9%)	450 (82.1%)	0.64 (0.49 to 0.83)	0.001	0.64 (0.49 to 0.83)	0.001	
Very remote Australia	1023		308 (30.1%)	715 (69.9%)	0.32 (0.27 to 0.39)	<0.001	0.32 (0.27 to 0.39)	<0.001	

* Missing data for 260 children

† Missing data for 18 children

## Discussion

Our study assessed the impact of a behaviour change intervention for health service providers in hospitals aimed at improving birth registrations, and hospital utilisation for Aboriginal babies in WA. After the implementation of our intervention, we found a 38% reduction in emergency presentations within 6 months of birth for babies that received the intervention. Although there was little evidence of an improvement on the proportion of births registered, hospital admissions within 3 or 6 months or emergency department presentations within 3 months of birth, all effects showed a decreasing direction. For babies born during our study period, 17.6% still had an unregistered birth 18 months post-implementation. Factors that were associated with not registering babies included babies who were low birth weight, having three or more children in the family and having the first antenatal visit after the first trimester. Living in the metropolitan area or in a high socioeconomic area was associated with babies having their births registered.

Based on the changes to the original protocol, our intervention was adapted to ensure improved information sharing and that it was embedded into the health system. Through a regular interview and feedback process among staff, and between staff and community, behaviours related to information and support that is provided to mothers can be evaluated, affirmed and updated, similar to a continuous quality improvement process.^40 41^ Ultimately, if a mother has some enhanced knowledge of important aspects of infancy and enhanced willingness to engage with primary care in the infancy period, there is reduced risk for emergency presentations and hospitalisations. Through the interviews and discussions with health professionals about important and imperative information sharing plus improved discharge planning and sharing of discharge summaries with chosen primary care providers, this would naturally facilitate continued thought and behaviour change to improve practice. As there were no secular trends in hospital admission or emergency presentation or seasonal differences, the improvements in emergency presentations at 6 months and general improvement in 3-month emergency presentations and up to 6-month hospitalisations are likely due to our intervention.

We saw modest changes in birth registrations as a result of the intervention. Despite this, we found that 17.6% of births were unregistered. It is unlikely that what became our intervention was not targeted enough to improve this outcome. Indeed, our original intent was to support mothers to fill in birth registration forms and help families to navigate the process of how to register their babies. This process would have likely had a more direct impact on this outcome. Previous research on birth registration of Aboriginal babies has found that 11% of Aboriginal births in WA were unregistered in children <16 years old in a birth cohort between 1980 and 2010.^12^ Factors associated with unregistered children included mothers who were aged <20 years old and those who lived in remote and very remote areas. Although our study found similar results such as living in a remote area being indicative of not having registered a baby, we found other important factors that promoted birth registrations including having the first antenatal visits after the first trimester. An important finding that has changed over time is that younger mothers were more likely to register their babies. Although our study was primarily focused on care after birth, we recognise the importance of high-quality antenatal care on not only the health and well-being of babies and their mothers but that continuity of care can also improve outcomes such as birth registration. There has been substantial work by individual Aboriginal Community Controlled Health Services to improve this outcome, including initiatives such as the Our Kids Count campaign, Pathfinders National Aboriginal Birth Certificate Program and other grassroots events aimed at supporting birth registration.^42 43^ We believe other initiatives such as working with families as they attend government-based child health checks and playgroups may be a feasible option to improving this outcome. In addition, the WA government has ongoing access to birth registration and midwives’ notification data. There is the opportunity for the government to determine the families that need the additional support and help them to register their babies during child health checks and fill this gap.

There are several limitations of this study. Due to the change in study design, there was an element of self-selection by hospitals of inclusion into the study. To determine whether this had an effect on our results, we investigated the trend of our outcomes occurring in a cohort of babies that were not born in intervention hospitals. However, a limitation of our data was that we were unable to complete this for birth registrations as we were only provided with year of registration and not exact dates. Due to only five hospitals being involved in the intervention, we had a reduced sample size which affected our power analysis and ability to see any changes that may have occurred. Our analyses also did not allow us to isolate the specific causal effect of the intervention. In addition, we did not collect data on what health service providers completed in hospital as part of the intervention. This limits our conclusion to the specific changes that occurred with maternity wards that may have influenced our outcomes. Birth registrations were recorded up to the end of 2019. Only data on year of birth registration were provided; therefore, we were not able to determine timeliness or capture those who may have been registered beyond this date. Despite these limitations of the data, the last babies born in the study were given 18 months for their birth to be registered. Area level Index of Relative Socio-Economic Disadvantage and the remoteness index, both from the 2016 Australian Bureau of Statistics’ Census of Population and Housing, were based on the mother’s address at the birth of the child which can cause misclassification when applied at an individual level.^44^ Strengths of this study include a large cohort of babies and use of data considered to be highly accurate.^38 45^ The Midwives’ Notification System has been reported to have a very high level of completion and clinical certainty.^46^

## Conclusions

Our study aimed to evaluate the impact of a behaviour change intervention for health service providers in Western Australian hospitals, targeting the improvement of birth registrations and hospital utilisation for Aboriginal babies. While our intervention led to a notable 38% reduction in emergency presentations within 6 months for babies who received it, we observed more modest improvements in birth registrations, hospital admissions within 3 and 6 months of birth and emergency department presentations within 3 months of birth.

The persistence of unregistered births, particularly among babies with low birth weight, in families with three or more children, and those with delayed first antenatal visits, underscores the challenges in addressing this issue comprehensively. In addition, the implementation of our intervention faced significant challenges during the pilot study, necessitating adaptations to ensure integration into the health system. Despite encountering obstacles, our improvement approach involving regular interviews and feedback among staff and the community showed positive results, especially in the reduction of emergency presentations.

Our study identifies the complexities surrounding birth registrations and improved hospital utilisations for Aboriginal babies, the importance of targeted interventions and ongoing efforts needed to address this issue comprehensively. Future endeavours should consider refining strategies based on the identified challenges and building upon the collaborative approaches between healthcare providers and the community.

## Supplementary material

10.1136/bmjopen-2024-089098 online supplemental file 1

## Data Availability

Data may be obtained from a third party and are not publicly available.
