# Supplementary material for: Outcomes following a behaviour change intervention within hospitals to improve birth registrations and hospital utilisation for Aboriginal and/or Torres Strait Islander infants: a quasi-experimental and cohort study
Source: BMJ Open. 2025 Oct 20;15(10):e089098. doi: 10.1136/bmjopen-2024-089098 (PMC12542530; doi:10.1136/bmjopen-2024-089098)
Supplement: online supplemental file 1 [file bmjopen-15-10-s001.docx]

Table of Contents

[Appendix A: Quasi-experimental design checklist 2](#_Toc205908086)

[Appendix B: Timing of intervention and sample size for each hospital and birth registration status 3](#_Toc205908087)

[Appendix D: Directed acyclic graph for birth registrations 7](#_Toc205908088)

[Appendix E: Timing of intervention and sample size for hospital admissions within 3 and 6 months of birth by hospital 8](#_Toc205908089)

[Appendix F: Timing of intervention and sample size for emergency department presentations within 3 and 6 months of birth by hospital 9](#_Toc205908090)

[Appendix G: Secular trends in hospital admissions and emergency department presentations 10](#_Toc205908091)

[Appendix H: Intervention sensitivity analysis 11](#_Toc205908092)

# Appendix A: Quasi-experimental design checklist

| **Checklist Question** | | **Answer (Yes/No)** |
| --- | --- | --- |
| 1. Was the intervention/comparator: | | |
|  | allocated to (provided for/administered to/chosen by) individuals? | No |
|  | allocated to (provided for/administered to/chosen by) clusters of individuals?^a^ | No |
|  | clustered in the way it was provided (by practitioner or organizational unit)?^b^ | Yes |
| 2. Were outcome data available: | | |
|  | after intervention / comparator only (same individuals)? | No |
|  | after intervention/comparator only (not all same individuals)? | Yes |
|  | before (once) AND after intervention/comparator (same individuals)? | No |
|  | before (once) AND after intervention/comparator (not all same individuals)? | Yes |
|  | multiple times before AND multiple times after intervention/comparator (same individuals)? | No |
|  | multiple times before AND multiple times after intervention/comparator (not all same individuals)? | Yes |
| 3. Was the intervention effect estimated by: | | |
|  | change over time (same individuals at different time-points)? | No |
|  | change over time (not all same individuals at different time-points)? | No |
|  | difference between groups (of individuals or clusters receiving either intervention or comparator)^c^? | Yes |
| 4. Did the researchers aim to control for confounding (design or analysis): | | |
|  | using methods that control in principle for any confounding? |  |
|  | using methods that control in principle for time invariant unobserved confounding? |  |
|  | using methods that control only for confounding by observed covariates? |  |
| 5. Were groups of individuals or clusters formed by: | | |
|  | randomization? | No |
|  | quasi-randomization? | Yes |
|  | explicit rule for allocation based on a threshold for a variable measured on a continuous or ordinal scale or boundary (in conjunction with identifying the variable dimension, below)? | No |
|  | some other action of researchers? | No |
|  | time differences? | No |
|  | location differences? | No |
|  | healthcare decision makers/practitioners? | No |
|  | participants’ preferences? | No |
|  | policy maker? | No |
|  | on the basis of outcome?^e^ | No |
|  | some other process? (specify) | No |
| 6. Were the following features of the study carried out after the study was designed: | | |
|  | characterization of individuals/clusters before intervention? | No |
|  | actions/choices leading to an individual/cluster becoming a member of a group?^e^ | No |
|  | assessment of outcomes? | No |
| 7. Were the following variables measured before intervention: | | |
|  | potential confounders? | Yes |
|  | outcome variable(s)? | Yes |

# Appendix B: Timing of intervention and sample size for each hospital and birth registration status

| **Site** | **Control dates** | **Interviews completed** | **Intervention dates** | **No. of control infants** | **No. of intervention infants** | **Total infants** | **Total control births registered (%)** | **Total intervention births registered (%)** |
| --- | --- | --- | --- | --- | --- | --- | --- | --- |
| Hospital 1 | 01/01/2016 – 30/06/2016 | July 2016 | 01/07/2016 – 31/12/2016 | 83 | 65 | 148 | 68 (81.9%) | 54 (83.1%) |
| Hospital 2 | 01/05/2016 – 31/10/2016 | Nov 2016 | 01/11/2016 – 30/04/2017 | 51 | 66 | 117 | 42 (82.4%) | n/p^a^ |
| Hospital 3 | 01/05/2017 – 31/10/2017 | Nov 2017 | 01/11/2017 – 30/04/2018 | 46 | 38 | 84 | n/p^a^ | 29 (76.3%) |
| Hospital 4 | 01/07/2017 – 31/12/2017 | Jan 2018 | 01/01/2018 – 30/06/2018 | 38 | 50 | 88 | n/p^a^ | 41 (82.0%) |
| Hospital 5 | 01/07/2017 – 31/12/2017 | Jan 2018 | 01/01/2018 – 30/06/2018 | 8 | 13 | 21 | n/p^a^ | n/p^a^ |
| *Total intervention sites* |  |  |  | *226* | *232* | *458* | *191 (84.5%)* | *193 (83.2%)* |

^a^ suppressed due to small cell sizes.

# Appendix C: Example report form interviews during the intervention

Responses from the interviews have been grouped into broad themes and listed under current practice, barriers, enablers and recommendations. NOTE: Hospital identifying information has been removed

**Current practice**

| Broad Theme | Detail |
| --- | --- |
| Family education | - Education about baby, mothers and father (at all times of contact) - Purple book and discharge packs available as a guide to discharge planning. |
| Encourage link with PHC, follow up referrals if required | - Identify the timing of visits and when to link with PHC |
| Early discharge | - Early discharge if healthy then onto home visiting - Encourage new mums to stay for 3 days but pushed out if beds required. |
| Discharge planning | - Follow and complete a special follow up refer to other organisations if required - Discharge planning from admission |
| Confirm family/community support network for mother | - Need to ensure family support is available if as often they do not get visited for a day or two. |
| Communication | - Communicate to all involved in a timely manner |

**Barriers**

| Broad Theme | Detail |
| --- | --- |
| Record keeping | - Contacts not current or incorrect |
| Time | - Lack of time to deliver education and implement best practice - Often checklist is not completed due to lack of time and patients leaving early - Time poor but this is not a good enough reason to not give mothers equal education and support – we have to address that. |
| Professional development | - Need education and professional development opportunities made more available for Midwives and other staff - Professional development and education is not supported by employers - Lack of cultural information |
| Management and staffing issues | - Lack of management understanding of issues - Staffing issues impacting on care and the ability to do more than the minimum |
| Fragmented discharge planning and connection to PHC | - Fragmented public health hospital based system of discharge planning - Connection with PHC services after discharge is often not coordinated well. |
| Fragmented family education – key information missed | - Early discharge if healthy then onto home visiting - Same midwife does labouring and postnatal care. Labouring has priority. - There needs to be more information available to the mothers on who to contact after discharge. - Conflicting and confusing information given - Lack of cultural information |
| Postnatal period education | - Postnatal period education is often not a focus when it should be - No standardised postnatal policies or procedures throughout Australia |
| Limited staff induction | - Lack of induction or in-service for new or junior staff. Best described as orientation and focused on labour (other information assumed) - No orientation or induction to service guidelines - Lack of cultural information |
| Transport | - No transport |
| Confirm family/community support network for mother | - Need to ensure family support is available if as often they do not get visited for a day or two. - Discharging back to remote areas |
| Family experience | - Negative perceptions of service organisation and reason for service |

**Enablers**

| Broad Theme | Detail |
| --- | --- |
| Care planning and culturally appropriate care | - Good care planning - Continuity of culturally care model needs to be developed. This should commence at the first antenatal visit and finish when the infant is 3 months of age. - Discharge planning should start at the first appointment and involve the mother and family |
| Consistency across the sector (care planning, information provided) | - There needs to be consistent across sector and evidenced based guidelines for best practice discharge planning, post-natal care and health education developed |
| Improved communication and links between tertiary and primary care | - Strong communication and links to PHC also with crisis care - Needs better feedback from PHC after handing over care to PHC - Standardised form to assist communication |
| Professional Development and enhanced cultural safety training options for staff | - Need education and professional development made more available for Midwives and other staff - Provide culturally specific training opportunities |
| Smartphone App e.g. purple book | - Smartphone apps |
| Enhanced support | - Focus on the first 6 weeks of life then its child and community health role - Need to ensure family support is available if as often they do not get visited for a day or two. - Home based education, home visits or walk in clinic important - Community follow up and care needs to be adapted to the context of home life - Include indigenous specific information in the baby magazine - Work with mothers whose birth plan doesn’t go to plan - Evidenced based consistent information given to mothers |
| Family-led care | - Better consumer input into care. |
| Liaison staff | - Liaison staff employed – independent agency |
| Increased regional based services | - More regional based services |

**Recommendations**

| Broad Theme | Detail |
| --- | --- |
| Enhanced support | - Prefill Medicare/registration forms as much as possible before giving to families. - Enhance support systems for families/mothers |
| Family Experience | - Facilitate a culturally safe birthing environment e.g. allowing 10+ people to support in the birthing suit - Alert mothers/families that Community Health are going to visit and/or allow Community Health access to the ward to introduce themselves. |
| Care planning and culturally appropriate care | - Enhanced information sharing between Maternity and Community Health, for example, Maternity confirm with Community Health which mothers are regional and whether they are staying in town prior to going home. This would allow Community Health to visit within the first few days of birth, - Continuity of care e.g. endorse midwives to work across AMS and hospital |
| Enhance communication across the health sector | - Shared communication system across sector 🡪 web based |
| Professional Development and enhanced cultural safety training options for staff | - Localised cultural training |
| Time | - Conduct an audit of day to day proactive to find out where/why midwives are time-poor |

# Appendix D: Example intervention logic model

# Appendix E: Directed acyclic graph for birth registrations

| 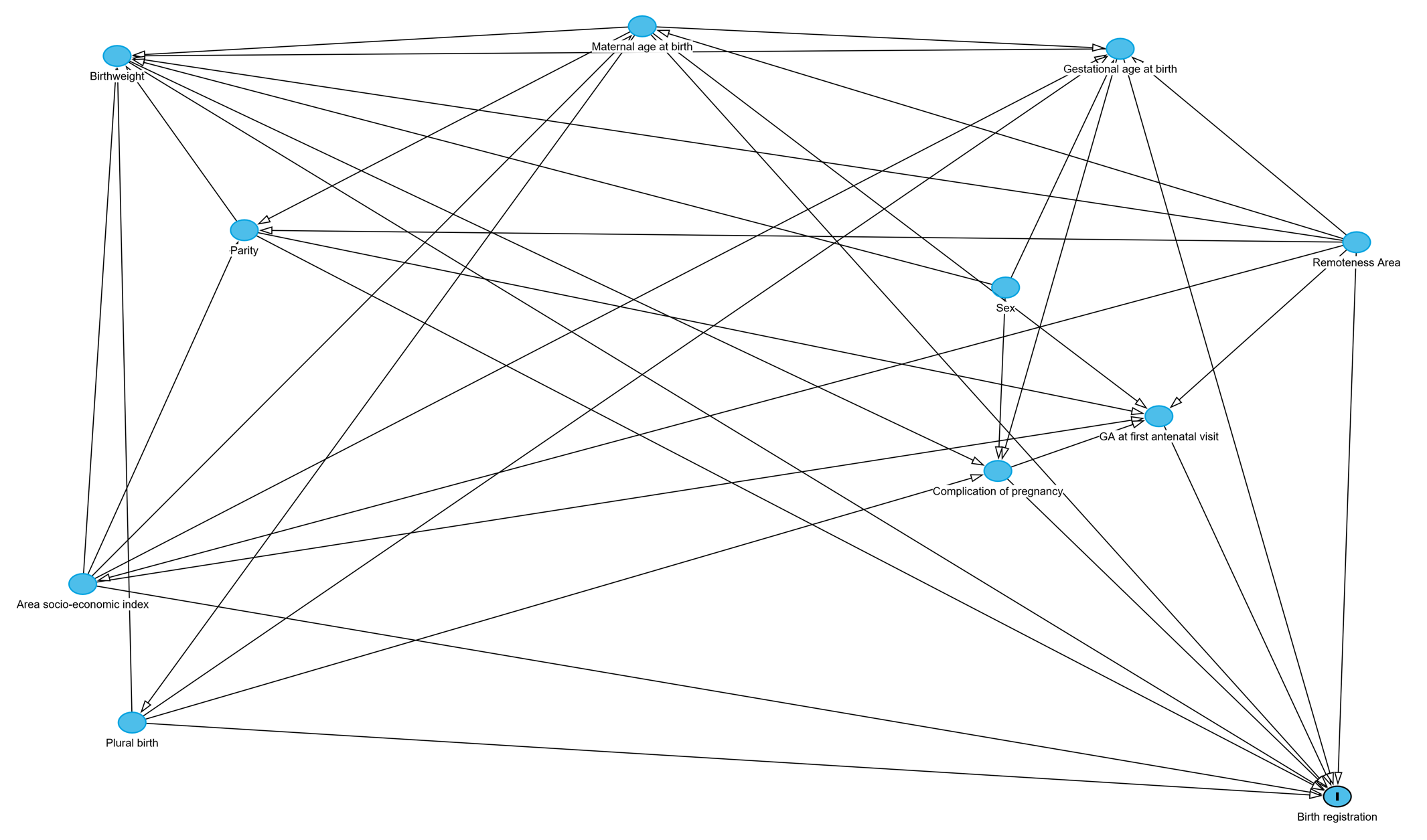 |
| --- |

# Appendix F: Timing of intervention and sample size for hospital admissions within 3 and 6 months of birth by hospital

| **Site** | **Control dates** | **Interviews completed** | **Intervention dates** | **No. of control infants** | **No. of intervention infants** | **Total infants** | **Total control admitted to hospital (%)** | **Total intervention admitted to hospital (%)** |
| --- | --- | --- | --- | --- | --- | --- | --- | --- |
| **Admission within 3 months of birth** | | | | | | | | |
| Hospital 1 | 01/01/2016 – 30/06/2016 | July 2016 | 01/07/2016 – 31/12/2016 | 83 | 65 | 148 | 14 (16.9%) | 11 (16.9%) |
| Hospital 2 | 01/05/2016 – 31/10/2016 | Nov 2016 | 01/11/2016 – 30/04/2017 | 51 | 66 | 117 | 12 (23.5%) | 9 (13.6%) |
| Hospital 3 | 01/05/2017 – 31/11/2017 | Nov 2017 | 01/11/2017 – 30/04/2018 | 46 | 38 | 84 | n/p^a^ | 9 (23.7%) |
| Hospital 4 | 01/07/2017 – 31/12/2017 | Jan 2018 | 01/01/2018 – 30/06/2018 | 38 | 50 | 88 | 11 (28.9%) | n/p^a^ |
| Hospital 5 | 01/07/2017 – 31/12/2107 | Jan 2018 | 01/01/2018 – 30/06/2018 | 8 | 13 | 21 | n/p^a^ | n/p^a^ |
| *Total* |  |  |  | *226* | *232* | *458* | *45 (19.9%)* | *36 (15.5%)* |
| **Admission within 6 months of birth** | | | | | | | | |
| Hospital 1 | 01/01/2016 – 30/06/2016 | July 2016 | 01/07/2016 – 31/12/2016 | 83 | 65 | 148 | 22 (26.5%) | 16 (24.6%) |
| Hospital 2 | 01/05/2016 – 31/10/2016 | Nov 2016 | 01/11/2016 – 30/04/2017 | 51 | 66 | 117 | 15 (29.4%) | 14 (21.2%) |
| Hospital 3 | 01/05/2017 – 31/11/2017 | Nov 2017 | 01/11/2017 – 30/04/2018 | 46 | 38 | 84 | n/p^a^ | n/p^a^ |
| Hospital 4 | 01/07/2017 – 31/12/2017 | Jan 2018 | 01/01/2018 – 30/06/2018 | 38 | 50 | 88 | 13 (34.2%) | 11 (22.0%) |
| Hospital 5 | 01/07/2017 – 31/12/2107 | Jan 2018 | 01/01/2018 – 30/06/2018 | 8 | 13 | 21 | n/p^a^ | n/p^a^ |
| *Total* |  |  |  | *226* | *232* | *458* | *58 (25.7%)* | *52 (22.4%)* |

^a^ suppressed due to small cell sizes.

# Appendix G: Timing of intervention and sample size for emergency department presentations within 3 and 6 months of birth by hospital

| **Site** | **Control dates** | **Interviews completed** | **Intervention dates** | **No. of control infants** | **No. of intervention infants** | **Total infants** | **Total control emergency department presentations (%)** | **Total intervention emergency department presentations (%)** |
| --- | --- | --- | --- | --- | --- | --- | --- | --- |
| **Presentation within 3 months of birth** | | | | | | | | |
| Hospital 1 | 01/01/2016 – 30/06/2016 | July 2016 | 01/07/2016 – 31/12/2016 | 83 | 65 | 148 | 28 (33.7%) | 20 (30.8%) |
| Hospital 2 | 01/05/2016 – 31/10/2016 | Nov 2016 | 01/11/2016 – 30/04/2017 | 51 | 66 | 117 | 28 (54.9%) | 32 (48.5%) |
| Hospital 3 | 01/05/2017 – 31/11/2017 | Nov 2017 | 01/11/2017 – 30/04/2018 | 46 | 38 | 84 | 26 (56.5%) | 18 (47.4%) |
| Hospital 4 | 01/07/2017 – 31/12/2017 | Jan 2018 | 01/01/2018 – 30/06/2018 | 38 | 50 | 88 | n/p^a^ | n/p^a^ |
| Hospital 5 | 01/07/2017 – 31/12/2107 | Jan 2018 | 01/01/2018 – 30/06/2018 | 8 | 13 | 21 | n/p^a^ | n/p^a^ |
| *Total* |  |  |  | *226* | *232* | *458* | *95 (42%)* | *84 (36.2%)* |
| **Presentation within 6 months of birth** | | | | | | | | |
| Hospital 1 | 01/01/2016 – 30/06/2016 | July 2016 | 01/07/2016 – 31/12/2016 | 83 | 65 | 148 | 49 (59%) | 35 (53.8%) |
| Hospital 2 | 01/05/2016 – 31/10/2016 | Nov 2016 | 01/11/2016 – 30/04/2017 | 51 | 66 | 117 | n/p^a^ | 39 (59.1%) |
| Hospital 3 | 01/05/2017 – 31/11/2017 | Nov 2017 | 01/11/2017 – 30/04/2018 | 46 | 38 | 84 | 31 (67.4%) | n/p^a^ |
| Hospital 4 | 01/07/2017 – 31/12/2017 | Jan 2018 | 01/01/2018 – 30/06/2018 | 38 | 50 | 88 | 16 (42.1%) | 18 (36%) |
| Hospital 5 | 01/07/2017 – 31/12/2107 | Jan 2018 | 01/01/2018 – 30/06/2018 | 8 | 13 | 21 | n/p^a^ | n/p^a^ |
| *Total* |  |  |  | *226* | *232* | *458* | *141 (62.4%)* | *117 (50.4%)* |

^a^ suppressed due to small cell sizes.

# Appendix H: Secular trends in hospital admissions and emergency department presentations

There is no evidence of secular trends affecting hospitalisations or emergency department presentations within this sample. The proportion of children using emergency or hospitals within 3 and 6 months of birth showed little to no change between January 2016 to June 2018 (OR: hospitalisation within 3 months 1.01, 95% CI 1.00 to 1.02; hospitalisation within 6 months 0.99, 95% CI 0.99 to 1.00; OR: emergency presentation within 3 months 1.00, 95% CI 1.00 to 1.01; OR: emergency presentation within 6 months 1.00, 95% CI 0.99 to 1.01; Units: months since January 2016).

Includes babies from all other WA birth hospitals that were not part of the intervention or control group (n=4,347)

# Appendix I: Sensitivity analyses

Washout period analysis
We completed a sensitivity analysis, conditional logistic regression on the effect of the intervention on birth registration, hospital admissions and emergency department presentations with 1-month washout period. There were no changes in the outcomes.

**Table I.1: New dates for intervention period with one month washout period**

| **Site** | **Control dates** | **Interviews completed** | **Intervention dates** | **New Intervention dates** |
| --- | --- | --- | --- | --- |
| Hospital 1 | 01/01/2016 – 30/06/2016 | July 2016 | 01/07/2016 – 31/12/2016 | 01/08/2016 – 31/01/2017 |
| Hospital 2 | 01/05/2016 – 31/10/2016 | Nov 2016 | 01/11/2016 – 30/04/2017 | 01/12/2016 – 31/05/2017 |
| Hospital 3 | 01/05/2017 – 31/10/2017 | Nov 2017 | 01/11/2017 – 30/04/2018 | 01/12/2017 – 31/05/2018 |
| Hospital 4 | 01/07/2017 – 31/12/2017 | Jan 2018 | 01/01/2018 – 30/06/2018 | 01/02/2018 – 31/07/2018 |
| Hospital 5 | 01/07/2017 – 31/12/2107 | Jan 2018 | 01/01/2018 – 30/06/2018 | 01/02/2018 – 31/07/2018 |

**Table I.2: Sensitivity analysis for the effect of the intervention on birth registration, hospital admissions and emergency department presentations**

|  | **Total number of infants** | **Number of infants with an event (%)** | **Unadjusted OR (95% CI)** | **p-value** |  |  |
| --- | --- | --- | --- | --- | --- | --- |
| *Birth registration* | | | | |  |  |
| Control | 226 | 191 (84.5%) | Ref. |  |  |  |
| Intervention | 230 | 195 (84.8%) | 1.00 (0.60 to 1.66) | 0.991 |  |  |
| *Hospital admissions within 3 months of birth* | | | | |  |  |
| Control | 226 | 45 (19.9%) | Ref. |  |  |  |
| Intervention | 230 | 35 (15.2%) | 0.72 (0.44 to 1.18) | 0.191 |  |  |
| *Hospital admissions within 6 months of birth* | | | | |  |  |
| Control | 226 | 58 (25.7%) | Ref. |  |  |  |
| Intervention | 230 | 53 (23.0%) | 0.87 (0.57 to 1.34) | 0.525 |  |  |
| *Emergency department presentations within 3 months of birth* | | | | |  |  |
| Control | 226 | 95 (42.0%) | Ref. |  |  |  |
| Intervention | 230 | 80 (34.8%) | 0.73 (0.50 to 1.08) | 0.117 |  |  |
| *Emergency department presentations within 6 months of birth* | | | | |  |  |
| Control | 226 | 141 (62.4%) | Ref. |  |  |  |
| Intervention | 230 | 114 (49.6%) | 0.61 (0.41 to 0.89) | 0.011 |  |  |

Seasonal analysis

We completed a sensitivity analysis on whether winter may influence a babies hospital admissions and emergency presentations.

**Table I.2: Sensitivity analysis for the effect of winter on emergency department presentations**

|  | **Total number of infants** | **Number of infants with an event (%)** | **OR (95% CI)** | **p-value** | **aOR* (95% CI)** | **p-value** |  |  |
| --- | --- | --- | --- | --- | --- | --- | --- | --- |
| *Hospital admissions within 3 months of birth* | | | | |  |  |  |  |
| Control | 226 | 45 (19.9%) | Ref. |  | Ref. |  |  |  |
| Intervention | 232 | 36 (15.5%) | 0.74 (0.46 to 1.20) | 0.227 | 0.77 (0.47 to 1.26) | 0.301 |  |  |
| *Hospital admissions within 6 months of birth* | | | | |  |  |  |  |
| Control | 226 | 58 (25.7%) | Ref. |  | Ref. |  |  |  |
| Intervention | 232 | 52 (22.4%) | 0.84 (0.54 to 1.29) | 0.426 | 0.85 (0.55 to 1.30) | 0.433 |  |  |
| *Emergency department presentations within 3 months of birth* | | | | |  |  |  |  |
| Control | 226 | 95 (42%) | Ref. |  | Ref. |  |  |  |
| Intervention | 232 | 84 (36.2%) | 0.78 (0.53 to 1.15) | 0.208 | 0.82 (0.60 to 0.1.22) | 0.325 |  |  |
| *Emergency department presentations within 6 months of birth* | | | | |  |  |  |  |
| Control | 226 | 141 (62.4%) | Ref. |  | Ref. |  |  |  |
| Intervention | 232 | 117 (50.4%) | 0.62 (.042 to 0.90) | 0.014 | 0.62 (.042 to 0.90) | 0.014 |  |  |

*adjusted for winter; aOR: adjusted odds ratio
