# Supplementary material for: Outcomes following a behaviour change intervention within hospitals to improve birth registrations and hospital utilisation for Aboriginal and/or Torres Strait Islander infants: a quasi-experimental and cohort study
Source: BMJ Open. 2025 Oct 20;15(10):e089098. doi: 10.1136/bmjopen-2024-089098 (PMC12542530; doi:10.1136/bmjopen-2024-089098)
Supplement: Supplementary file 2 [file bmjopen-2024-089098.reviewer_comments.pdf]

PEER REVIEW HISTORY

BMJ Open publishes all reviews undertaken for accepted manuscripts. Reviewers are asked to complete a checklist review form and are provided with free text boxes to elaborate on their assessment. These free text comments are reproduced below.

ARTICLE DETAILS

Title (Provisional)

Outcomes following a behaviour change intervention within hospitals to improve birth registrations and hospital utilisation for Aboriginal and/or Torres Strait Islander infants: a quasi-experimental and cohort study

Authors

McAuley, K.; Strobel, Natalie Ann; Christensen, Daniel; Edmond, Karen Margaret; Jacoby, Peter; McAullay, Daniel

---

VERSION 1 - REVIEW

**Reviewer**	**1**
**Name**	**Dack, Kyle**
**Affiliation**	**University of Bristol, MRC Integrative Epidemiology Unit**
**Date**	**23-Sep-2024**
**COI**	**None**

This RCT manuscript used appropriate statistical methods and in most cases explained the analyses and decision-making with a good level of detail. However, the statistical review is incomplete because the main tables were missing from the manuscript. Additionally, the secondary model of 11 covariates may not be suitable for minimizing the risk of confounding for all covariates. Please see below for specifics:

Abstract

1. The objectives state that the abstract is given to hospital staff, and in the participants section the control/intervention groups are described in terms of number of babies. It is difficult for the abstract reader to easily see how these link together. A sentence explaining that children born in each hospital post-intervention were counted as in the intervention group would help.

2. The participants section states 4537 babies were included in the cohort analysis, but in the results 4573.

Methods

3. P7 lines 7-9. 25 hospitals were allocated to clusters developed based on 22 distinct areas - but later in the methods it is stated that the 25 hospitals were randomly allocated to clusters. It may be clearer if there is a single section/paragraph explaining the clustering design and allocation.

4. P7 line 20. Suggest rephrasing to “five hospitals, one randomly selected from each cluster” to make it clearer that each cluster was sampled - if this is the case?

5. Figure 1. Suggest changing “excluded (n = 20 hospitals)” to “randomly excluded”, so readers do not confuse this as being due to an exclusion criteria.

6. P7 line 50. It would be helpful to reference the protocol which includes additional details of the intervention.

7. How long did the intervention take in each hospital?

8. The intervention is defined as starting on the first date of the month of the intervention. Appendix B does not give exact dates of the intervention - were the interventions all began at the start of the month, or were there cases where the intervention actually occurred mid-month? Is there a risk of some children being classed as in the treatment group when in fact they were controls? If so, the authors could consider a sensitivity analysis excluding those born in the month of the intervention. More details of this would be informative.

9. P8 line 16. I suggest rephrasing this to make the timing clearer, including what happened near to the intervention: babies born within the window 6 months to 1 day (?) prior to the intervention were classified as controls, and those born 1 day (?) after the intervention until 6 months after were classified as the treatment group.

10. P9 line 12. Please state how covariates were identified - were all theorized to have possible links to registration probability?

11. Could the authors please explain the rationale for categorizing age.

12. P10 lines 43-51. Many of the 11 covariates seem likely to be correlated or causal with one another. Could the authors please include an explanation of what they considered the confounding variables to likely be. Is the single model likely to produce an association at low risk of confounding for all 11 covariates? It seems that there is a risk of some covariates mediating the association of other covariates - which should be avoided as it would divide the effect of interest (see https://www.ncbi.nlm.nih.gov/pmc/articles/PMC7850592/) and bias the results towards a null finding. It may be that different models are needed to accurately estimate the association for different covariates. A DAG may help the authors decide this.

Results

13. The main text tables seem to be missing (and therefore not reviewed).

14. Appendix F. Most of the trend looks as if it is attributable to the final three quarters of the study. Registrations were recorded until 18 months later. Was this enough time to capture the full "tail" of registrations? This could be checked by looking at the births in 2016 and whether many took longer than 18 months. This would strengthen the result and could be stated in the discussion.

Reviewer **2**

Name **Martiniuk, Alexandra**

Affiliation **University of Sydney Sydney Medical School, School of Public Health**

Date **04-Nov-2024**

COI **None**

BMJ Review Nov 4 2024

I thank the authors for their research, aiming to improve the registration of Aboriginal and/or Torres Strait Islander babies and reduce their ED and hospital admissions. This publication reports upon two related studies: stated as a cluster RCT and cohort in 5 hospitals in WA, Australia. The intervention was delivered to health workers, the outcomes observed for babies. Control babies were born ½ year before the intervention. Factors influencing birth registration was a secondary objective (this was the cohort study).

This study found reduced ED presentations for 'intervention' babies, but no other improvements. The cohort found LBW babies, and late first antenatal visit were less likely to be birth registered.

This study was funded by NHMRC.

Comments:

Title – having infants children in the title is not ideal – suggest write one or the other

Abstract:

“Results: There was evidence of a 38% reduction in emergency presentations within six months for babies who received the intervention”

Edit please – babies did not receive the intervention – the health workers at the hospital where the babies were born received the intervention. Noting also that Aboriginal colleagues and community members often don't like the use of the word 'intervention'.

The methods for the cluster RCT could be made clearer in the abstract

The manuscript is somewhat hard to read due to it reporting on two study designs and changes in planned study design.

The intervention description could be made more clear. I think this was the intervention: “interview process with health service providers, with feedback from research staff, to enable a behaviour change intervention that focussed on discharge planning, providing information to mothers, and improving standard practice by health service providers.”

This lacked detail

A logic model (theory of change) here would assist in aligning intervention components with anticipated outcomes

Later in the manuscript (page 6) “Utilising feedback from hospital staff who participated in the pilot study it was determined that an interview process with health service providers, and feedback from research staff, enabled behavioural change in discharge planning, information provided to mothers, and standard practice among the health service providers.”

Further detail is required -what behavioural change in discharge planning (what changed? What changes were desired?), what information to mothers and when and why….and what does ‘standard practice’ mean here?

The intervention was given to staff in 5 hospitals, and the babies in these hospitals were followed for outcomes (registration, ED, hosp) and the controls in this cluster RCT were who? -babies from the other/aggregated data from other hospitals or also intervention hospitals 6 mos prior to intervention? Not clear in the manuscript.

Methods

CONSORT statement use is useful (page 5)– but there is no mention of using the CONSORT extension for cluster RCTs though the study design is stated as a cluster RCT. Albeit not a typical one as this was originally planned as a stepped wedge design.

I appreciated the authors' transparent reporting about the change in sample recruitment and study design.

Unclear the time period when the intervention was delivered. Important since the protocol paper was published about 10 years ago.

This manuscript will benefit from further peer-review by a statistician familiar with stepped-wedge cluster RCTs, as well as cluster RCTs.

Is sample size sufficient now that the study is no longer a stepped-wedge design? A larger sample size typically needed for cluster RCT (versus RCT and versus stepped-wedge cluster RCTs).

I'm not clear the terminology for the final study design is most appropriate – as cluster RCTs typically randomise clusters to receive an intervention, and compare to clusters not receiving an intervention (randomisation by cluster) – and outcomes are determined AT THE INDIVIDUAL level.

I understand the intervention hospitals had data at the individual level, but the comparator/control was an aggregate? (from: "As data were aggregated for all hospitals other than the intervention sites" page 9)

Measurement of outcomes – I found this confusing in the manuscript

Page 6 "The aim of the intervention during the study was to facilitate behaviour change in healthcare workers"

Was behaviour change in health workers measured?

Page 7 "This may have occurred through mother knowledge etc etc ".....describes the mechanism by which changes in health worker behaviour may have influenced the outcomes of "birth registration, hospital admissions, and emergency department presentations." were these mechanisms measured? Perhaps a diagram will help.

Data analysis section:

re

"As data were aggregated for all hospitals other than the intervention sites, intervention analyses were restricted to the five intervention sites. Given no substantial differences at baseline between the intervention and control group we did not adjust the analyses"

I understand there are debates re this -see Adjustment for Baseline Characteristics in Randomized Clinical Trials | Research, Methods, Statistics | JAMA | JAMA Network

Also page 9 – analytic approach would benefit from further statistical peer-review. Logistic regression is mentioned as the analytic approach however I query whether mixed-effects models or hierarchical models may be needed, or other adaptations to manage the mix of individual (intervention hospitals) and aggregate (control hospitals) data, as well as clustered

nature of the data. There is no mention of the ICC, or differences between intervention clusters, or size of clusters.

Typos

STRENGTHS AND LIMITATIONS • 82.4% (3769/4573) of birth Aboriginal babies –remove word 'birth'

Figure 1

25 hospitals were randomised

Then 5 allocated

**This part needs greater clarity. I understand there were logistical challenges and hence the decision to have 5 hospitals. But the randomisation here is confusing. 25 hospitals were randomised -to what is not specified? I assume about 12 to control and 12 to intervention (and an additional 1 to?)

Then 5 hospitals allocated. 5 hospitals from which randomised arm? This needs clarity?

Figure shows 5 hospitals allocated, but insufficient detail re intervention and control – how many hospitals to intervention (3?) and control (2?). Or 5 randomised to intervention and then other hospitals aggregated data were considered the controls? This needs further explanation.

I'm not clear if randomisation is appropriately used in this study or its analyses – perhaps further detail will be useful.

Appendix B

Now I see the controls in the RCT were babies at each hospital born 6 months prior to the intervention. Was this for all hospitals (aggregate controls and intervention hospitals?) This was not fully clear in the manuscript writing

Discussion

Reductions in ED presentations? Of which presentations/categories?

Real life solutions to the existing problems influencing birth registration?

Birth record exists via hospital, but registration has not occurred – perhaps an intervention supporting those mothers who did not register the birth (due to cost, complexity, time) would be worth evaluating next?

Trial publication was 2016, this submission is nearly 10 years later. No action items just an observation.

Patient and public involvement -disappointing that the mothers couldn't be involved more. More detail on the partnership with ACCHOs would be useful.

VERSION 1 - AUTHOR RESPONSE

Reviewer Feedback	Notes
Reviewer 1	Thank you for reviewing our journal submission
1. the statistical review is incomplete because the main tables were missing from the manuscript	Thank you for letting us know. We apologise for not submitting them. We have now provided these tables.
2. Abstract: The objectives state that the abstract (intervention?) is given to hospital staff, and in the participants section the control/intervention groups are described in terms of number of babies. It is difficult for the abstract reader to easily see how these link together. A sentence explaining that children born in each hospital post-intervention were counted as in the intervention group would help.	We have revised the abstract so that it's clear that only babies in the five hospitals were included in the analysis.
3. Abstract: The participants section states 4537 babies were included in the cohort analysis, but in the results 4573.	We have fixed this typo.
4. Methods: P7 lines 7-9. 25 hospitals were allocated to clusters developed based on 22 distinct areas - but later in the methods it is stated that the 25 hospitals were randomly allocated to clusters. It may be clearer if there is a single section/paragraph explaining the clustering design and allocation.	We have now revised the 'Randomisation' heading to 'Randomisation and allocation to intervention'. We have provided more information.
5. Methods: P7 line 20. Suggest rephrasing to "five hospitals, one randomly selected from each cluster" to make it clearer that each cluster was sampled - if this is the case?	We have now clarified this statement:<br><br>'As a result, a total of five hospitals, one from each from each cluster participated in the intervention'
6. Methods: Figure 1. Suggest changing "excluded (n = 20 hospitals)" to "randomly excluded", so readers do not confuse this as being due to an exclusion criteria.	We have revised Figure 1 and have added 'randomly excluded'
7. Methods: P7 line 50. It would be helpful to reference the protocol which includes additional details of the intervention.	We have revised our methods and now referenced the protocol to refer people to the original intervention. This information is provided under the heading 'Original Study'.
8. Methods: How long did the intervention take in each hospital?	It is unclear how long health service providers would have supported mothers in each hospital that received the intervention. As the intervention was aimed at

	supporting health service providers to change how they delivered care and provide better support to mothers it would be dependent on what support mothers needed. These data were not collected as part of the trial. We have added this information to the methods section and as a limitation of the trial.
9. Methods: The intervention is defined as starting on the first date of the month of the intervention. Appendix B does not give exact dates of the intervention - were the interventions all began at the start of the month, or were there cases where the intervention actually occurred mid-month? Is there a risk of some children being classed as in the treatment group when in fact they were controls? If so, the authors could consider a sensitivity analysis excluding those born in the month of the intervention. More details of this would be informative.	Appendix C gives exact dates that the intervention was rolled out. However, we agree that there could be some risk of babies being classed as the intervention instead of the control. We have completed a sensitivity analysis with a one month wash-out period. We have provided details in the 'Data Analysis' section and in Appendix H. There were no changes in the outcomes.
10. Methods: P8 line 16. I suggest rephrasing this to make the timing clearer, including what happened near to the intervention: babies born within the window 6 months to 1 day (?) prior to the intervention were classified as controls, and those born 1 day (?) after the intervention until 6 months after were classified as the treatment group.	We have now refined the intervention dates as:<br><br>'The intervention was defined as commencing the first day of the month that interviews were completed and followed on for 6 full calendar months (Appendix C). Babies born one day before the intervention and the 6 calendar months prior to the intervention in each intervention hospital were to be part of the control group.'
11. Methods: P9 line 12. Please state how covariates were identified - were all theorized to have possible links to registration probability?	Covariates are based on those variables that could influence whether babies' births were registered. We have now completed a DAG to provide a more robust understanding of what covariates are potential confounders.
12. Methods: Could the authors please explain the rationale for categorizing age.	In general, the basis for categorising as opposed to using a continuous variable would be if either we were concerned there was a non-linear association or the categories are of a priori interest.

	In the case of maternal age at birth, there is a substantial literature on teenage motherhood, and we elected to follow this interest.
13. Methods: P10 lines 43-51. Many of the 11 covariates seem likely to be correlated or causal with one another. Could the authors please include an explanation of what they considered the confounding variables to likely be. Is the single model likely to produce an association at low risk of confounding for all 11 covariates? It seems that there is a risk of some covariates mediating the association of other covariates - which should be avoided as it would divide the effect of interest (see https://www.ncbi.nlm.nih.gov/pmc/articles/PMC7850592/) and bias the results towards a null finding. It may be that different models are needed to accurately estimate the association for different covariates. A DAG may help the authors decide this.	To reduce bias, we agree that completing a DAG will reduce these effects. As such we have completed a DAG and added it to the appendix. We have changed aOR in Table 4 to reflect this. Only the results for mother's age changed as a result of the new analysis.
14. Results: The main text tables seem to be missing (and therefore not reviewed).	Thank you for letting us know. We apologise for not submitting them. We have now provided these tables.
15. Results: Appendix F. Most of the trend looks as if it is attributable to the final three quarters of the study. Registrations were recorded until 18 months later. Was this enough time to capture the full "tail" of registrations? This could be checked by looking at the births in 2016 and whether many took longer than 18 months. This would strengthen the result and could be stated in the discussion.	Thank you for raising this point. We have investigated this concern and have found that we are unable to provide a definitive answer about the 'tail' in registrations. Unfortunately, we were unable to receive the date of birth registration and we only provided the year of registration. As a result, we can only crudely estimate the time it has taken for babies to have their births registered. As a result, we have decided to remove the sensitivity analysis and the use of year in birth as an exposure in our cohort study. We have also added this in as a limitation of our data in the discussion.
Reviewer 2	Thank you for reviewing our journal submission
16. Title: having infants children in the title is not ideal – suggest write one or the other	We have now clarified and changed to 'infants' only.
17. Abstract: "Results: There was evidence of a 38% reduction in emergency presentations within six months for babies who received the intervention"	We have revised this sentence to:<br><br>'There was evidence of a 38% reduction in emergency

Edit please – babies did not receive the intervention – the health workers at the hospital where the babies were born received the intervention. Noting also that Aboriginal colleagues and community members often don't like the use of the word 'intervention'.	presentation within 6 months of birth for babies who were in hospital during the intervention period (OR 0.62, 95% CI 0.42 to 0.91; Table 2).'<br><br>This project's lead CI is Aboriginal (DM) and is also the last author on the paper. The team have been working with health services to improve the quality of care to Aboriginal children and families for many years and are aware of the nuances around some words. We are comfortable in keeping 'intervention' in the manuscript.
18. Abstract: The methods for the cluster RCT could be made clearer in the abstract	We have now revised the study design to be a quasi-experimental design and refer to this throughout the paper.
19. The manuscript is somewhat hard to read due to it reporting on two study designs and changes in planned study design.	We have revised the methods of the manuscript to make this clearer.
20. The intervention description could be made more clear. I think this was the intervention: "interview process with health service providers, with feedback from research staff, to enable a behaviour change intervention that focussed on discharge planning, providing information to mothers, and improving standard practice by health service providers."<br>This lacked detail<br>A logic model (theory of change) here would assist in aligning intervention components with anticipated outcomes<br>Later in the manuscript (page 6) "Utilising feedback from hospital staff who participated in the pilot study it was determined that an interview process with health service providers, and feedback from research staff, enabled behavioural change in discharge planning, information provided to mothers, and standard practice among the health service providers."<br>Further detail is required -what behavioural change in discharge planning (what changed? What changes were desired?), what information to mothers and when and why….and what does 'standard practice' mean here?	We have now added more details to the intervention section of the methods. We have not completed a logic model as this was not done at the time of the changes in the intervention.
21. The intervention was given to staff in 5 hospitals, and the babies in these hospitals were followed for outcomes (registration, ED, hosp) and the controls in this cluster RCT were who? -babies from the other/aggregated data from other hospitals or also intervention hospitals 6 mos prior to	We have now revised the methods section and Figure 1. We have also added more details under the 'Randomisation and allocation to the intervention'.

intervention? Not clear in the manuscript.	
22. Methods: CONSORT statement use is useful (page 5)– but there is no mention of using the CONSORT extension for cluster RCTs though the study design is stated as a cluster RCT. Albeit not a typical one as this was originally planned as a stepped wedge design.	We have removed the CONSORT statement as reviewers have correctly pointed out this did not end up being a cluster-RCT. As a result, it is not appropriate to report this information. We have added information about the study design through a quasi-experimental checklist which is provided in Appendix A.
23. Methods: Unclear the time period when the intervention was delivered. Important since the protocol paper was published about 10 years ago.	We have added this information about the dates under the 'Intervention and control groups' heading with specific dates for each hospital provided in Appendix C.
24. Methods: This manuscript will benefit from further peer-review by a statistician familiar with stepped-wedge cluster RCTs, as well as cluster RCTs.<br>Is sample size sufficient now that the study is no longer a stepped-wedge design? A larger sample size typically needed for cluster RCT (versus RCT and versus stepped-wedge cluster RCTs).<br>I'm not clear the terminology for the final study design is most appropriate – as cluster RCTs typically randomise clusters to receive an intervention, and compare to clusters not receiving an intervention (randomisation by cluster) – and outcomes are determined AT THE INDIVIDUAL level.<br>I understand the intervention hospitals had data at the individual level, but the comparator/control was an aggregate? (from: "As data were aggregated for all hospitals other than the intervention sites" page 9)	This was a failed cluster-RCT and is now considered to be a quasi-experimental study design. As such we have not analysed the data as a cluster-RCT. The authorship team have three people (PJ, DM and NS) with degrees in statistics and we spent substantial time determining the most robust method to analyse the data.
25. Methods: Measurement of outcomes – I found this confusing in the manuscript	It is unclear what section you are referring to, however, we have been through the information under the 'Outcomes' heading and removed information that is not directly relevant to birth registrations.
26. Methods: Page 6 "The aim of the intervention during the study was to facilitate behaviour change in healthcare workers"<br>Was behaviour change in health workers measured?	No behaviour change was not measured in the health workers. We have added this as a limitation to the intervention.
27. Page 7 "This may have occurred through mother knowledge etc etc "…..describes the mechanism by which changes in health worker behaviour may have influenced the outcomes of "birth registration, hospital admissions, and emergency department presentations." were these	See also comment 12

mechanisms measured? Perhaps a diagram will help.	
28. Data analysis section: "As data were aggregated for all hospitals other than the intervention sites, intervention analyses were restricted to the five intervention sites. Given no substantial differences at baseline between the intervention and control group we did not adjust the analyses"<br>I understand there are debates re this -see Adjustment for Baseline Characteristics in Randomized Clinical Trials | Research, Methods, Statistics | JAMA | JAMA Network	The data has been analysed using a conditional logistic regression which aims to remove any time-invariant (fixed) measured or unmeasured effects at the hospital level. Although we have assessed differences between the control and intervention group there is still a possibility of unmeasured confounding. Our study design is also at risk of secular bias that may differentiate the two groups, however, as seen this has not been seen for hospitalisation and emergency presentations. We have also completed a sensitivity analysis using a one-month washout. This would also highlight if there were any changes within the groups that might lead to biases. However, none were seen. We are confident that it is appropriate that we have not adjusted out results.
29. Data analysis section: Also page 9 – analytic approach would benefit from further statistical peer-review. Logistic regression is mentioned as the analytic approach however I query whether mixed-effects models or hierarchical models may be needed, or other adaptations to manage the mix of individual (intervention hospitals) and aggregate (control hospitals) data, as well as clustered nature of the data. There is no mention of the ICC, or differences between intervention clusters, or size of clusters.	To determine the effects of the intervention we completed a conditional logistic regression that removed any time-invariant (fixed) measured or unmeasured effects at the hospital level. This is an appropriate analysis given that we were not completing an analysis for a cluster-RCT.
30. Typos: STRENGTHS AND LIMITATIONS • 82.4% (3769/4573) of birth Aboriginal babies –remove word 'birth'	This information has been removed. We have been asked to revise this section. Please see comment 5.
31. Figure 1<br>25 hospitals were randomised<br>Then 5 allocated<br>**This part needs greater clarity. I understand there were logistical challenges and hence the decision to have 5 hospitals. But the randomisation here is confusing. 25 hospitals were randomised -to what is not specified? I assume about 12 to control and 12 to intervention (and an additional 1 to?)<br>Then 5 hospitals allocated. 5 hospitals from which randomised arm? This needs clarity?<br>Figure shows 5 hospitals allocated, but insufficient detail re	We have now re-drawn Figure 1 to better reflect the study design. We have also revised the methods to provide clarity around the randomisation process for both the original study and the study that was finally completed.

intervention and control – how many hospitals to intervention (3?) and control (2?). Or 5 randomised to intervention and then other hospitals aggregated data were considered the controls? This needs further explanation.<br>I'm not clear if randomisation is appropriately used in this study or its analyses – perhaps further detail will be useful.	
32. Appendix B<br>Now I see the controls in the RCT were babies at each hospital born 6 months prior to the intervention. Was this for all hospitals (aggregate controls and intervention hospitals?) This was not fully clear in the manuscript writing	We have revised the manuscript and methods to make this clearer.
33. Discussion: Reductions in ED presentations? Of which presentations/categories?	We maintained the outcomes from our original study which was all-cause hospitalisations and emergency department presentations. As such, we have not categorised these presentations.
34. Discussion: Real life solutions to the existing problems influencing birth registration?	We have added the following in the discussion section:<br><br>'We believe other initiatives such as working with families as they attend government-based child health checks and playgroups may be a feasible option to improving this outcome. In addition, the WA government has ongoing access to birth registration and midwives notification data. There is the opportunity for the government to determine the families that need the additional support and help them to register their babies during child health checks and fill this gap.'
35. Discussion: Birth record exists via hospital, but registration has not occurred – perhaps an intervention supporting those mothers who did not register the birth (due to cost, complexity, time) would be worth evaluating next?	We have added this into the discussion section:<br><br>'We believe other initiatives such as working with families as they attend government-based child health checks and playgroups may be a feasible option to improving this outcome. In addition, the WA government has ongoing access to birth registration and midwives

	notification data. There is the opportunity for the government to determine the families that need the additional support and help them to register their babies during child health checks and fill this gap.'
36. Discussion: Patient and public involvement - disappointing that the mothers couldn't be involved more. More detail on the partnership with ACCHOs would be useful.	Although ACCHOs aren't always directly involved with maternity wards in hospitals there are often staff that work across them. Staff from ACCHOs were interviewed and played a key role in developing the new intervention and informing hospitals of the barriers they faced. We have now added the following to this section:<br><br>'This included working with staff to design the new intervention and interviewing staff to help hospitals understand the barriers and facilitators that were experienced by families within organisations. Information from each intervention report was fed back to relevant ACCHOs.'

VERSION 2 - REVIEW

**Reviewer**	**1**
**Name**	**Dack, Kyle**
**Affiliation**	**University of Bristol, MRC Integrative Epidemiology Unit**
**Date**	**24-Feb-2025**
**COI**	

The authors have made extensive changes to the manuscript. The observational part of the study is now clearer and I have much more confidence that confounding bias has been minimized.

However, the quasi-experimental part of the study, while improved, lacks important details. In particular the description of the intervention is confusing and lacks transparency. The intervention involved interviews, feedback, and training to hospital staff. But throughout the paper it is framed as if families/babies received the intervention – they did not. The method

section describing the intervention lacks many details on the process and it is surprising to me that the authors replied that they do not know how long the intervention in each hospital took. This severely limits the replicability of the study, and possibly its clinical utility if we don't know what was done.

The duration of the intervention remain unclear to me, but there also seems to be a risk of bias from seasonal changes in infant emergency admissions? This should be discussed – does the change in admissions post intervention differ from local seasonal variation?

Abstract

1. In the abstract and elsewhere in the paper the phrasing "babies who received the intervention" is used. I agree with the second reviewer that this is not seem accurate – the intervention involved training hospital staff, and therefore the intervention was received by hospital staff / hospitals. This sentence "There was evidence of a 38% reduction in emergency presentations within six months for babies who received the intervention" may mislead readers because it implies some interaction with babies themselves. It is more accurate to report a "38% reduction in odds of emergency presentations for babies born to hospitals six months following the staff training".

Introduction

2. The second study aim has been revised, but is not clear to me. "reduce hospital admissions and emergency presentations for babies < 3 months and < 6 months old". Is "< 3 months" not redundant here, because all babies less than 3 months are also less than 6 months old. I think this needs rephrasing to clarify what is meant.

Methods

3. The authors did not give a clear response to my feedback "18. Methods: How long did the intervention take in each hospital?". The response stated it is unclear how long mothers received the intervention – but mothers didn't receive the intervention, staff did? The response leaves me uncertain regarding what the intervention actually involved, and it is surprising that a general estimate of the time duration cannot be produced.

4. P5 Line 49. "involved" should be "born before or after", the babies themselves did not participate or have any direct interaction as part of the behaviour change programme.

5. Study design: I suggest signposting that the second part of this is an observational analysis to achieve the secondary aims of the study, this will improve the clarity.

6. The randomization and allocation section describes randomly selecting five hospitals from twenty two. This section describes random sampling of hospitals to participate in the study, I don't think it describes randomization. All five hospitals received the intervention, and the control is before/after intervention. The subheading should be "hospital selection" or similar.

7. Page 7 line 26. The intervention was delivered between June 2016 and July 2018 – it is not clear what this means. In appendix Fig 1 the intervention is shown as a single day. There was

no intervention in 2018. I think this should be rephrased – data was collected between those dates?

8. The intervention is not described in enough details to really understand what happened. There are thorough details of staff interviews. A report was then produced for recommendations to improve – but what did this contain? What was the process for producing it? Staff then attended training with facilitators – what material was used, what were the instructions given to facilitators?

9. "We assumed that changes within the hospital process would occur over six months and as such considered changes to the intervention during this period." Appendix B figure 1 shows the intervention as only a single point in time, rather than a gradual process. This figure is very useful and I think it would help in the main paper.

10. Page 11 line 10. "each analysis was adjusted..". From my reading, there is only one multivariable model? So I think "each" should just be "the"?

Results

11. Table 1. Again I don't think it is clear to refer to babies as controls or intervention when they were not involved in it. Also not all of these are characteristics of the infant, some are characteristics of the mother, or the birth. "Births born before or after the intervention" seems more accurate.

12. Table 4. Columns 3 and 4 are already reported in Table 3. The final column is useful, but I suggest rephrasing to "Covariates to minimize confounding" or just "covariates". It would be clearer if a line, or bolding was used to distinguish between each model.

Reviewer 2

Name Martiniuk, Alexandra

Affiliation University of Sydney Sydney Medical School, School of Public Health

Date 26-Mar-2025

COI

Regarding authors' Response to #23

DAGs help determine causal effects, but they do not directly "reduce effects" unless you mean that proper adjustment based on the DAG can mitigate confounding bias?

#30

Response: "We have now added more details to the intervention

section of the methods. We have not completed a

logic model as this was not done at the time of the

changes in the intervention."

Although I agree that drafting a logic model prior to any intervention development, implementation or evaluation is the most effective approach – I still believe that presenting a logic model in the paper (or as a supplementary file) is useful for the following reasons:

a) Logic models help to conceptualize and visualize a theory of change – the assumptions about how the intervention is expected to work – and where the outcomes are anticipated to be observed

b) Having a logic model can help improve future program design and scale-up

---

VERSION 2 - AUTHOR RESPONSE

**Reviewer Feedback**	**Notes**
Reviewer 1	
1. The duration of the intervention remain unclear to me, but there also seems to be a risk of bias from seasonal changes in infant emergency admissions? This should be discussed – does the change in admissions post intervention differ from local seasonal variation?	We have now provided additional information regarding the intervention.<br><br>In regard to seasonal changes, we have In regard to seasonal changes, we have completed an additional analysis. Emergency presentation has been characterised as a binary outcome in our analysis. As a result, we needed to create an additional binary variable that would adequately reflect seasonality and add this as a confounding variable to our analysis. We defined this new variable 'winter' as babies who had the opportunity to present to emergency during winter if winter was within 0-92 days of their date of birth (3 months) or within 0-184 days of their date of birth (6 months). We used winter as the main season as this is the time that there is often an increase in emergency presentations due to respiratory infections (e.g. RSV, influence). Due to sample size we were only able to create a binary variable. Based on our analysis we found no changes to our outcome. This approach should control for any imbalances between the intervention and control groups in their exposure to

	ED presentations risks due to winter. We have added this information to the analysis section and reported the findings in the appendix.
2. Abstract: In the abstract and elsewhere in the paper the phrasing "babies who received the intervention" is used. I agree with the second reviewer that this is not seem accurate – the intervention involved training hospital staff, and therefore the intervention was received by hospital staff / hospitals. This sentence "There was evidence of a 38% reduction in emergency presentations within six months for babies who received the intervention" may mislead readers because it implies some interaction with babies themselves. It is more accurate to report a "38% reduction in odds of emergency presentations for babies born to hospitals six months following the staff training".	We agree that this should have been revised in our first review. We have now revised the sentence so that it does not imply that babies have received the intervention.
3. Introduction: The second study aim has been revised, but is not clear to me. "reduce hospital admissions and emergency presentations for babies < 3 months and < 6 months old". Is "< 3 months" not redundant here, because all babies less than 3 months are also less than 6 months old. I think this needs rephrasing to clarify what is meant.	We don't believe this is redundant. If we have only <6 months old as the aim that would imply that only one analysis was completed, which is not true. Two analyses were completed. We have left the aim as is.
4. Methods: The authors did not give a clear response to my feedback "18. Methods: How long did the intervention take in each hospital?". The response stated it is unclear how long mothers received the intervention – but mothers didn't receive the intervention, staff did? The response leaves me uncertain regarding what the intervention actually involved, and it is surprising that a general estimate of the time duration cannot be produced.	We have now provided an estimate of the time that hospital staff participated in the intervention.
5. Methods: P5 Line 49. "involved" should be "born before or after", the babies themselves did not participate or have any direct interaction as part of the behaviour change programme.	In this instance 'involved' is correct, as we are writing about the initial project which the intervention was delivered directly to mothers. We have added 'Aboriginal infants and their mothers' to clarify the unit that would receive the intervention.
6. Methods, Study design: I suggest signposting that the second part of this is an observational analysis to achieve the secondary aims of the study, this will improve the clarity.	We have now added to 'observational analysis' to the secondary aims in the abstract, at the end of introduction and as part of the results heading - 'Observational analysis of factors influencing birth registration.

7. Methods: The randomization and allocation section describes randomly selecting five hospitals from twenty two. This section describes random sampling of hospitals to participate in the study, I don't think it describes randomization. All five hospitals received the intervention, and the control is before/after intervention. The subheading should be "hospital selection" or similar.	We have now changed the heading in the methods to read 'Hospital selection'.
8. Methods: Page 7 line 26. The intervention was delivered between June 2016 and July 2018 – it is not clear what this means. In appendix Fig 1 the intervention is shown as a single day. There was no intervention in 2018. I think this should be rephrased – data was collected between those dates?	We have moved this Figure to the main document and it is now labelled as Figure 2. We have also changed the legend to 'Intervention implementation' to reflect that the intervention was delivered through this period rather than on one day.<br><br>This also reflects the text under the heading 'Intervention and control groups': The intervention was defined as commencing the first day of the month that interviews were completed and the following six full calendar months (Figure 2).
9. Methods: The intervention is not described in enough details to really understand what happened. There are thorough details of staff interviews. A report was then produced for recommendations to improve – but what did this contain? What was the process for producing it? Staff then attended training with facilitators – what material was used, what were the instructions given to facilitators?	We have added more detail of the intervention, reports and training that was provided.
10. Methods: "We assumed that changes within the hospital process would occur over six months and as such considered changes to the intervention during this period." Appendix B figure 1 shows the intervention as only a single point in time, rather than a gradual process. This figure is very useful and I think it would help in the main paper.	We have now added this figure into the main document and is referred to as Figure 2.
11. Methods: Page 11 line 10. "each analysis was adjusted..". From my reading, there is only one multivariable model? So I think "each" should just be "the"?	This is a redundant sentence as we are now using a DAG to assess for confounding. We have deleted this sentence.
12. Results: Table 1. Again I don't think it is clear to refer to babies as controls or intervention when they were not involved in it. Also not all of these are characteristics of the infant, some are characteristics of the mother, or the birth.	We have now revised the title of Table 1 to be:

"Births born before or after the intervention" seems more accurate.	Table 1: Characteristics of births born before and after the intervention
13. Results: Table 4. Columns 3 and 4 are already reported in Table 3. The final column is useful, but I suggest rephrasing to "Covariates to minimize confounding" or just "covariates". It would be clearer if a line, or bolding was used to distinguish between each model.	The raw numbers for Table 3 Col 3 and 4 are reported in Table 4. However, the percentages are different to reflect the OR in Table 4. We have not changed these data.<br><br>We have revised the heading in the last column in Table 4 to be 'Covariates'
Reviewer 2	
14. Regarding authors' Response to #23: DAGs help determine causal effects, but they do not directly "reduce effects" unless you mean that proper adjustment based on the DAG can mitigate confounding bias?	Thank you for the clarification and yes this is what we meant.
15. Response #30: "We have now added more details to the intervention section of the methods. We have not completed a logic model as this was not done at the time of the changes in the intervention."<br><br>Although I agree that drafting a logic model prior to any intervention development, implementation or evaluation is the most effective approach – I still believe that presenting a logic model in the paper (or as a supplementary file) is useful for the following reasons:<br>a) Logic models help to conceptualize and visualize a theory of change – the assumptions about how the intervention is expected to work – and where the outcomes are anticipated to be observed<br>b) Having a logic model can help improve future program design and scale-up	We have provided an example logic model in Appendix D and provided the following information under the 'Intervention and control groups' heading:<br><br>We have developed an indicative program logic to illustrate one example of the types of change the intervention sought to achieve (Appendix C). This example is intended to demonstrate the underlying theory of change, acknowledging that specific activities and intended outcomes vary across different implementation settings. The intervention is designed to be flexible so that activities can address context-specific service gaps and local needs while remaining aligned with the overall research program goals.

VERSION 3 - REVIEW

Reviewer **1**

Name **Dack, Kyle**

Affiliation University of Bristol, MRC Integrative Epidemiology Unit

Date 20-Aug-2025

COI

The authors have made significant clarifications which address all feedback I previously shared.
